# The first Miocene fossils from coastal woodlands in the southern East African Rift

**DOI:** 10.1016/j.isci.2023.107644

**Published:** 2023-08-15

**Authors:** René Bobe, Vera Aldeias, Zeresenay Alemseged, Robert L. Anemone, Will Archer, Georges Aumaître, Marion K. Bamford, Dora Biro, Didier L. Bourlès, Melissa Doyle Boyd, David R. Braun, Cristian Capelli, João d’Oliveira Coelho, Jörg M. Habermann, Jason J. Head, Karim Keddadouche, Kornelius Kupczik, Anne-Elisabeth Lebatard, Tina Lüdecke, Amélia Macôa, Felipe I. Martínez, Jacinto Mathe, Clara Mendes, Luis Meira Paulo, Maria Pinto, Darya Presnyakova, Thomas A. Püschel, Frederico Tátá Regala, Mark Sier, Maria Joana Ferreira da Silva, Marc Stalmans, Susana Carvalho

**Affiliations:** 1 Gorongosa National Park, Sofala, Mozambique; 2 Primate Models for Behavioural Evolution Lab, Institute of Human Sciences, School of Anthropology, University of Oxford, Oxford OX2 6PN, UK; 3 Interdisciplinary Center for Archaeology and Evolution of Human Behavior (ICArEHB), Universidade do Algarve, 8005-139 Faro, Portugal; 4 Department of Organismal Biology & Anatomy, University of Chicago, Chicago, IL 60637, USA; 5 University of North Carolina at Greensboro, Department of Anthropology, Greensboro, NC 27402-6170, USA; 6 Max Planck Partner Group, Department of Archaeology and Anthropology, National Museum, Bloemfontein, South Africa; 7 Department of Geology, University of the Free State, Bloemfontein, South Africa; 8 Centre Européen de Recherche et d'Enseignement de Géosciences de l'Environnement, CEREGE - UM 34 Aix-Marseille Université, CNRS, IRD, Collège de France, INRAE, OSU Institut Pythéas, Technopole Environnement Arbois - Méditerranée, Domaine du Petit Arbois, Avenue Louis Philibert, Les Milles-Aix en Provence BP80, 13545 AIX en Provence, Cedex 04, France; 9 Evolutionary Studies Institute and School of Geosciences, University of the Witwatersrand, Johannesburg, South Africa; 10 Department of Biology, University of Oxford, Oxford OX1 3RB, UK; 11 Department of Earth and Planetary Sciences, Rutgers University, Piscataway, NJ 08854, USA; 12 Center for the Advanced Study of Human Paleobiology, Department of Anthropology, George Washington University, Washington, DC 20052, USA; 13 Technological Primate Research Group, Max Planck Institute for Evolutionary Anthropology, Deutscher Platz 6, 04103 Leipzig, Germany; 14 Dipartimento delle Scienze Chimiche, della Vita e della Sostenibilità Ambientale, Università di Parma, 43124 Parma, Italy; 15 Centre for Functional Ecology, University of Coimbra, 3000-456 Coimbra, Portugal; 16 GeoZentrum Nordbayern, Friedrich-Alexander-Universität Erlangen-Nürnberg, 91054 Erlangen, Germany; 17 Department of Zoology, University of Cambridge, Cambridge CB2 3EJ, UK; 18 Departamento de Antropología, Facultad de Ciencias Sociales, Universidad de Chile, Santiago, Chile; 19 Emmy Noether Group for Hominin Meat Consumption, Max Planck Institute for Chemistry, 55128 Mainz, Germany; 20 Senckenberg Biodiversity and Climate Research Centre, 60325 Frankfurt, Germany; 21 Departamento de Arqueologia e Antropologia, Faculdade de Letras e Ciências Sociais, Universidade Eduardo Mondlane, Maputo, Mozambique; 22 Escuela de Antropología, Facultad de Ciencias Sociales, Pontificia Universidad Católica de Chile, Santiago, Chile; 23 AESDA – Associação de Estudos Subterrâneos e Defesa do Ambiente, Torres Vedras, Portugal; 24 CNRS Aix-Marseille Université, Marseille, France; 25 Department of Early Prehistory and Quaternary Ecology, University of Tübingen, 72074 Tübingen, Germany; 26 Ecology and Evolutionary Biology Division, School of Biological Sciences, University of Reading, Reading RG6 6LA, UK; 27 CENIEH, 09002 Burgos, Spain; 28 Department of Earth Sciences, Faculty of Geosciences, Utrecht University, Utrecht 3584 CS, the Netherlands; 29 CIBIO, Centro de Investigação Em Biodiversidade e Recursos Genéticos, InBIO Laboratório Associado, Campus de Vairão, Universidade do Porto, 4485-661 Vairão, Portugal; 30 BIOPOLIS Program in Genomics, Biodiversity and Land Planning, CIBIO, Campus de Vairão, 4485-661 Vairão, Portugal; 31 ONE - Organisms and Environment Group, Cardiff University, School of Biosciences, Sir Martin Evans Building, c5:15, Cardiff CF10 3AX, UK; 32 ASTER TEAM, CEREGE, 13545 Aix-en-Provence, France

**Keywords:** Geochemistry, Evolutionary biology, Forestry

## Abstract

The Miocene was a key time in the evolution of African ecosystems witnessing the origin of the African apes and the isolation of eastern coastal forests through an expanding arid corridor. Until recently, however, Miocene sites from the southeastern regions of the continent were unknown. Here, we report the first Miocene fossil teeth from the shoulders of the Urema Rift in Gorongosa National Park, Mozambique. We provide the first 1) radiometric ages of the Mazamba Formation, 2) reconstructions of paleovegetation in the region based on pedogenic carbonates and fossil wood, and 3) descriptions of fossil teeth. Gorongosa is unique in the East African Rift in combining marine invertebrates, marine vertebrates, reptiles, terrestrial mammals, and fossil woods in coastal paleoenvironments. The Gorongosa fossil sites offer the first evidence of woodlands and forests on the coastal margins of southeastern Africa during the Miocene, and an exceptional assemblage of fossils including new species.

## Highlights

• First Miocene fossil sites discovered in the southern East African Rift
• First cosmogenic nuclide dating of fossil sites in the region
• New sites fill major biogeographic gap in evolution of African ecosystems
• Multiple lines of evidence of ancient coastal forest and woodland ecosystems

## Graphical abstract

**Figure:**
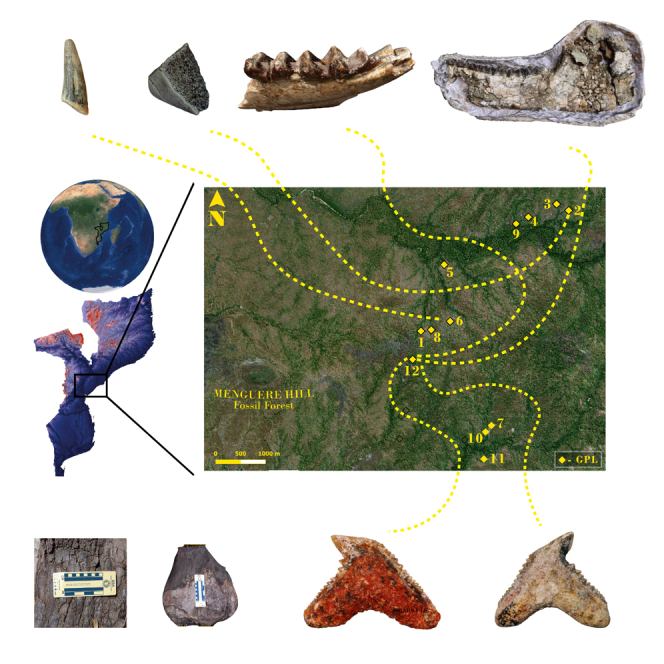

## Introduction

**Figure 1:**
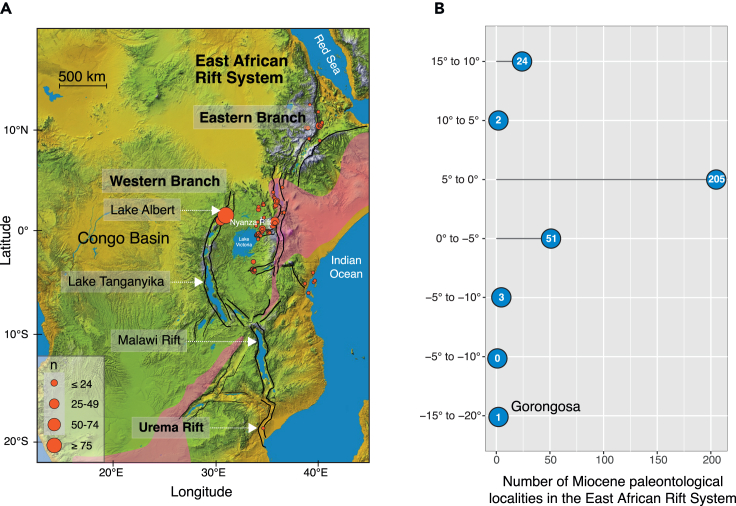
The East African Rift System (A) The East African Rift System (EARS) with the Eastern Branch, the Western Branch, and some of the major basins and rifts, including the Urema Graben at its southern end. The development of the EARS since the Miocene has played a major role in shaping the physical environments and modifying the conditions under which plants and animals have been evolving in eastern Africa. Shaded area depicts hypothetical extent of arid corridor during the Miocene. Base map from Nasa Shuttle Radar Topography Mission (https://www2.jpl.nasa.gov/srtm/). (B) Number of Miocene paleontological localities along the EARS by latitude. There are many Miocene localities in the rift near the equator, but the record away from the equator, especially to the south, is very sparse. Gorongosa is the only Miocene paleontological locality in the southern ∼1500 km of the EARS. Locality data from the Paleobiology Database https://paleobiodb.org/classic.

**Figure 2:**
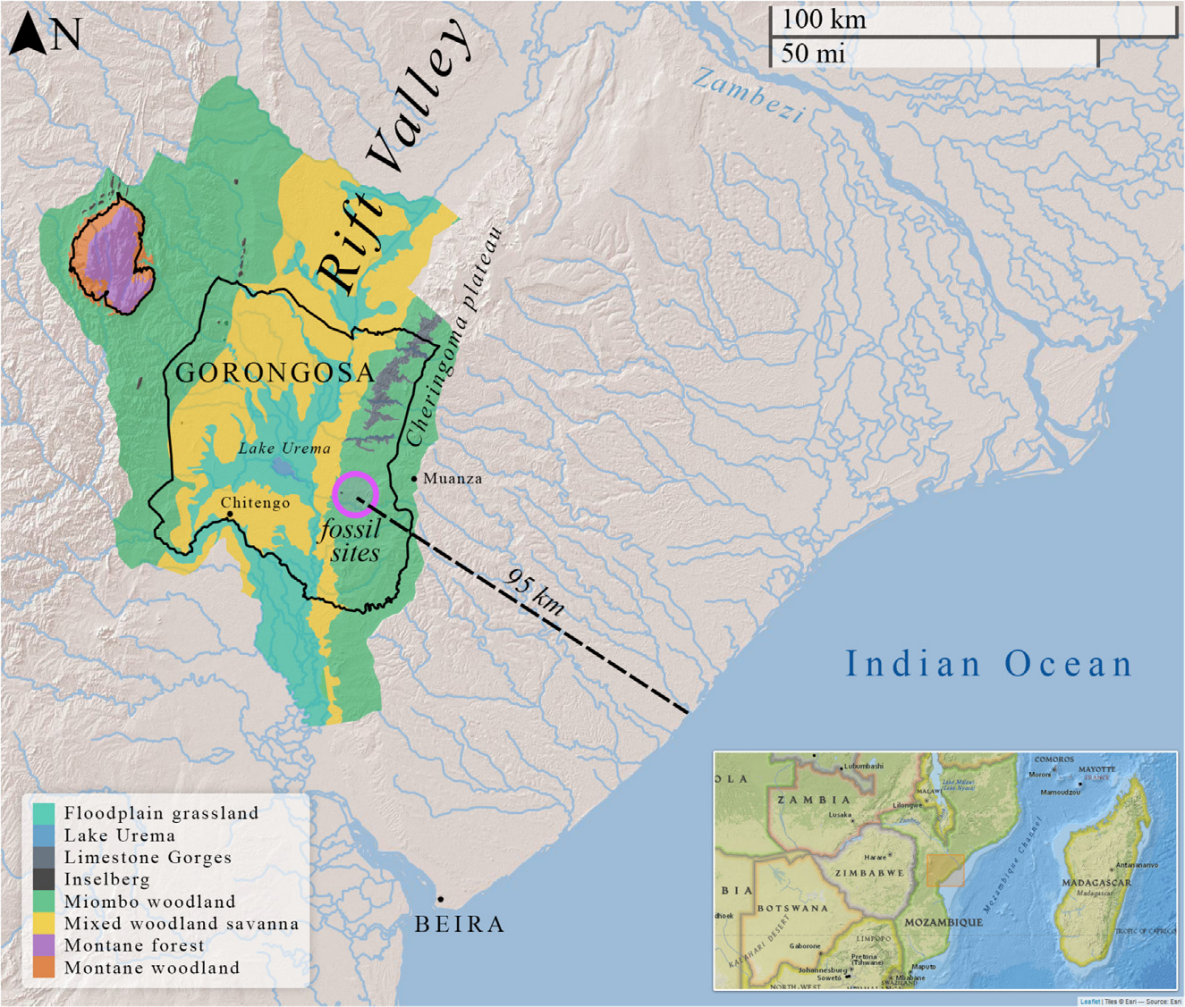
Map of Gorongosa National Park along the East African Rift Valley The park hosts a wide range of environments. The new paleontological sites on the Cheringoma Plateau are ∼95 km from the coast.

Much of our knowledge about African Miocene vertebrates and their environments derives from paleontological sites along the East African Rift System (EARS).^1^^,^^2^^,^^3^^,^^4^^,^^5^^,^^6^^,^^7^^,^^8^ However, considerable geographic and temporal gaps in the fossil record obscure a full appreciation of past biodiversity, biogeography, and ecosystem evolution on the continent. For example, until recently, there were no sites with Miocene mammals in the southern 1,500 km of the EARS (Figure 1). Thus, the Miocene faunas and ecosystems of this southern region have remained virtually unknown. Furthermore, none of the well-known Miocene fossil sites in the EARS provides evidence of eastern African coastal forests, a major ecosystem that may have played a key role in the evolution of several mammalian lineages.^9^^,^^10^ More broadly, in the context of southern Africa, there are only a few sites with terrestrial mammalian faunas, and the known sites (e.g., Berg Aukas, Namibia) are poorly contextualized.^11^^,^^12^^,^^13^ Although the necessity of documenting new fossil sites in previously unknown areas is widely appreciated and advocated,^14^^,^^15^^,^^16^ discovering entirely new paleontological beds is a rare event.^17^ Here, we describe the first dentognathic specimens of fossil vertebrates discovered in the East African Rift of central Mozambique. The specimens derive from the Mazamba Formation on the eastern shoulder of the Urema Rift in Gorongosa National Park (GNP) (Figure 2).^18^ Cosmogenic nuclide dating presented here indicates that the Gorongosa paleontological localities are of Miocene age. These localities formed under estuarine conditions and represent the first documentation of eastern African coastal forests in the Miocene. The emerging fossil record from Gorongosa opens the possibility of testing, for the first time, key hypotheses about an expanding northeast-southwest arid corridor that would have isolated the eastern coastal forests from those in the central parts of Africa, and for exploring the importance of these processes for hominid origins (Figure 1).^10^ Gorongosa Park is now well known for its successful wildlife restoration project,^19^ and these new paleontological sites in the park open a unique window on the fauna and environments of ancient Africa.

**Figure 3:**
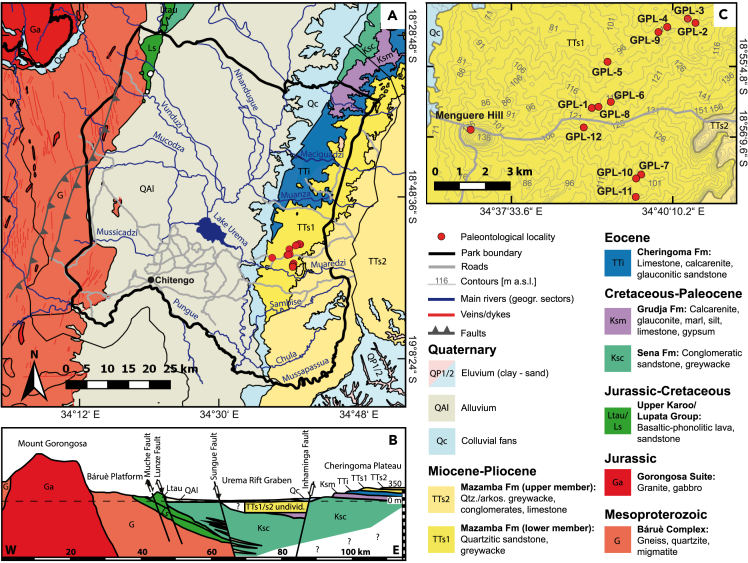
Gorongosa Paleontological Localities and geological formations (A) Geological map of Gorongosa National Park and surrounding areas. (B) Vertical geological cross section of the Urema Rift stretching from Mount Gorongosa to Inhaminga village. (C) Map section showing the locations of the fossiliferous sites (GPL = Gorongosa Paleontological Locality). Figure modified from Habermann et al.^18^ and references therein, with new paleontological localities added.

At the southern end of the EARS, the Urema Graben crosses Gorongosa along an approximately north-south axis, with the Cheringoma Plateau on the east and Mount Gorongosa dominating the northwestern region (Figures 2 and 3). The Urema Graben represents one of the youngest sections of the EARS.^20^^,^^21^ The eastern shoulder of the Urema Graben is the Cheringoma Horst, an uplifted block bounded by the Inhaminga Fault on the west between the Pungue and Zambezi Rivers.^22^ Several geological formations are exposed in the Cheringoma Plateau, including the Sena Formation (Cretaceous), the Grudja Formation (with late Cretaceous and early Tertiary levels), the Cheringoma Formation (Eocene nummulitic limestones), and the Mazamba Formation (Mazamba sands attributed to the Miocene)^22^^,^^23^ (Figure 3). The Mazamba Formation is named after exposures along the Mazamba River 25 km southwest of Inhaminga in the Cheringoma Plateau. At the type locality in the upper Mazamba River, this formation attains 140 m in thickness.^22^^,^^23^ These deposits are separated from the underlying Cheringoma Formation by a well-defined erosional unconformity resulting from marine regression. According to Flores (1973: 105),^22^ “There is an erosional unconformity between the Eocene and the Miocene, with no intervening Oligocene, indicating considerable uplift in post-Eocene-pre-Miocene times”. In the 1968 geological map of Mozambique, the Mazamba Formation is divided into two members separated by a chert horizon (as reproduced in Tinley 1977). The lower member (“grés de cor púrpura”, or purple clays/sands) (TT_S1_ in the 1968 geological map; Figure 3) is composed of purplish to reddish medium-grained argillaceous sands, which contain gastropods, bivalves, crustaceans, and foraminifera, and are interpreted to be littoral marine intercalated with deltaic deposits. The upper member (TT_S2_) is referred to as the Inhaminga beds (“camadas de Inhaminga”), composed of medium-to-coarse arkosic sands with some irregular conglomerate layers (Figure 3). Although there are some discrepancies and contradictions in the literature, most previous descriptions focused on the geology of the Cheringoma region consider the lower part of the Mazamba Formation to be of Miocene age and the upper part of the sequence to extend into the Mio-Pliocene.^18^^,^^23^^,^^24^^,^^25^^,^^26^ Thus, we use the term Mazamba Formation to refer to the Mazamba/Inhaminga sequence in the Cheringoma Horst, with two informal members, a lower member and an upper member separated by a chert horizon. In the field, we identified the nodular chert layer separating the lower and upper sequences and undertook geological and paleontological surveys of both lower and upper deposits.

The dating of this sedimentary sequence has been hampered by the lack of radio-isotopic age determinations. Neogene volcanism has been less intensively developed in the southern EARS than in regions to the north (e.g., Afar, Main Ethiopian Rift, Omo-Turkana Basin, Kenya Rift), and volcanic ash layers amenable to radiometric dating seem to be rare. In a regional context, recent research on the Zambezi Delta by Ponte and colleagues has identified a major unconformity at the end of the Oligocene related to uplift of the South African Plateau, with the “Mazamba sands” deposited above this unconformity during the early Miocene (Aquitanian and Burdigalian stages).^27^

## Results

During the 2016–2019 field seasons, the Paleo-Primate Project Gorongosa discovered and documented seven paleontological localities with fossil vertebrates: GPL-1, GPL-2, GPL-6, GPL-7, GPL-8, GPL-11, and GPL-12. Three additional localities produced invertebrates only (GPL-3, GPL-9, and GPL-10), and two yielded *ex situ* stone tools (GPL-4 and GPL-5). Menguere Hill, with abundant fossil wood, is the westernmost fossiliferous locality and it is not identified by a GPL number (Figure 3). These localities are listed in Table 1. Here, we provide new data and integrate several lines of evidence from the Mazamba Formation, including 1) sedimentology and depositional environments of the fossil localities, 2) radiometric age determinations based on cosmogenic nuclides, 3) stable isotopes from pedogenic carbonates, 4) paleobotanical remains, and 5) vertebrate paleontology.

**Table 1:** Gorongosa paleontological localities (GPLs) and depositional environments

Locality	Elev in m	Facies	Depositional environments	Notes
GPL-1	112	Conglomerate, sandstones, claystones, marlstones	Fluvial to estuarine	Abundant vertebrate fossils
GPL-2	120	Sandstones, claystone	Estuarine to shallow marine	Crustaceans, gastropods, bivalves
GPL-3	116	Sandstones, claystone	Estuarine to shallow marine	Crustaceans, gastropods, bivalves
GPL-4	110	Conglomerates, quartzitic sandstone	Fluvial?	Surface stone tools (not *in situ*)
GPL-5	99	Conglomerates, quartzitic sandstone	Fluvial?	Surface stone tools (not *in situ*)
GPL-6	115	Sandstones, claystones	Fluvial to estuarine, marine?	Large mammal bones
GPL-7	101	Siliciclastic sandstone, pebble lag	Fluvial	Mammal maxillary fragment
GPL-8	111	Conglomerate, sandstones	Fluvial, reworked estuarine/marine	Striostera margaritacea oyster
GPL-9	107	Conglomerate, sandstones	Fluvial, reworked estuarine/marine	Mollusks, red algae, serpulid
GPL-10	99	Sandstones	Coastal delta plain	Oysters, bivalves, crustaceans
GPL-11	100	Rudstone, sandstones	Shallow marine	Abundant oysters, gastropods
GPL-12	114	Sandstones, claystones	Fluvial to estuarine	Abundant *in situ* vertebrates
Menguere Hill	108	Calcrete, silcrete	Paleo-pan	Fossil wood, tree trunks
Mussapassua	160	Coarse quartzitic sands	Fluvial	Upper member Mazamba Fm

### Sedimentology and stratigraphy of the lower Mazamba Formation

**Figure 4:**
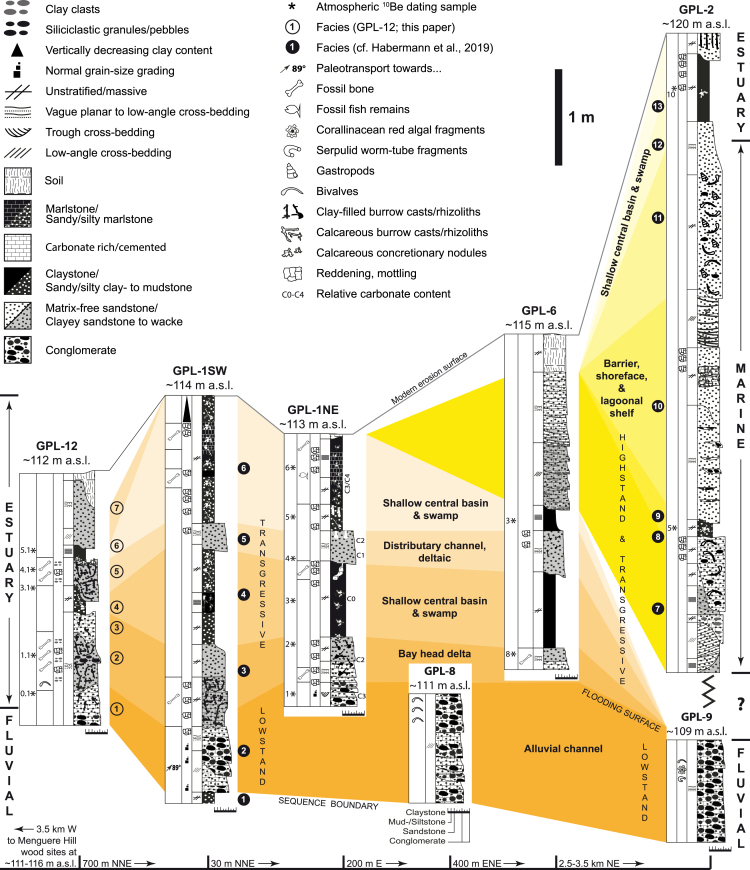
Stratigraphic sections Modified and updated from Habermann et al.^18.^

Based on regional stratigraphic relationships, sedimentary facies, facies architecture, and the emerging fossil record, Habermann and colleagues^18^ interpreted the sedimentary successions of the lower member of the Mazamba Formation exposed in the study region as representing a paleoenvironmental mosaic of estuarine and riverine forest/woodland systems. Estuarine sequences accumulated prior to rifting as compound incised-valley fills on a low-gradient coastal plain following transgression, receiving continental sediment from source terranes west of today’s Urema Graben. The lower Mazamba succession at the southwestern paleontological sites (GPL-1, GPL-6, GPL-7, GPL-8, GPL-12, see Figure 3) is dominated by basal conglomeratic and sandy facies overlain by clayey sandstones to wackes and sandy clay and marlstone units (Figure 4). These successions are interpreted as lowstand (fluvial) and transgressive (estuarine) assemblages, comprising alluvial channel, bay head delta, shallow central basin or swamp, and fluvio-deltaic distributary channel facies from base to top. In contrast, the northeastern localities represent laterally correlative (GPL-9) as well as younger stratigraphic levels (GPL-2, GPL-3); they are sand dominated and contain marine invertebrates and some fossil mammals. These successions are interpreted as transgressive highstand assemblages consisting of barrier, shore-face, and lagoonal shelf facies.

GPL-1 and GPL-12 are the most fossiliferous localities. The sedimentary sequence of GPL-1 was described in detail by Habermann et al.,^18^ and here we describe the sedimentary succession of GPL-12 (Figures 3 and 4). The gully sidewall at GPL-12 exposes a 3 m thick section comprising seven distinct sedimentary facies. Coarse, granule-, and pebble-bearing quartz sandstones that are moderately cemented by carbonate and contain variable amounts of clay, clayclasts, mottling, and bioturbation form the base of the succession (Facies 1–3). Bedding, occasionally picked out by pebble stringers or abrupt vertical grain-size changes, is only poorly developed. A single cast of a fossil bivalve was found in Facies 2 close to the bottom of the section. Mottling, reddish discoloration, and clay-filled bioturbation casts, including *Thalassinoides* isp., are most common in Facies 2. This facies yielded numerous vertebrate fossils including mandibles from various taxa as well as isolated teeth and bone fragments. Brown, sandy claystones with sand-filled bioturbation casts (Facies 4) follow above, which in turn are overlain by clayey sandstones of Facies 5 that include the second level in the section with large fossil vertebrate remains. At and near the top surface of Facies 5, carbonate accumulated in the form of finely distributed powder, as small concretionary nodules, or as thin crusts, suggesting a disconformity surface. A thin band of olive green to reddish waxy claystone follows next (Facies 6), which is overlain by medium-grained, well-sorted sandstones that are cross-bedded in places. In Figure 4, we present tentative correlations between localities based on lithological and sedimentological criteria.

Grain-size and sorting characteristics of the basal sandstones of Facies 1–3 suggest a fluvial depositional environment. The vertebrate, invertebrate, and trace fossils in this part of the succession, however, comprise terrestrial and potentially brackish or marine elements (bivalve in Facies 2 as well as *Thalassinoides* isp., most commonly produced by burrowing decapod crustaceans). The fossil remains thus refine paleoenvironmental inferences, suggesting fluvio-deltaic conditions, possibly in a river-dominated estuarine context (bay-head delta assemblage). Fossil preservation and abundance in Facies 2 may suggest high sedimentation rates and relatively rapid burial, perhaps during a storm or flood event. Claystone units in the GPL-12 succession may indicate overbank or mudpond deposition in a fluvio-deltaic environment or may reflect a deepening trend so that estuarine muds formed under brackish to marginal marine conditions following transgression.

### Cosmogenic nuclides - atmospheric ^10^Be dating

To establish a chronology for the Mazamba Formation, we applied the authigenic ^10^Be/^9^Be cosmogenic nuclide dating method, hereafter referred to as atmospheric ^10^Be dating, since the method is based on the atmospherically produced isotope ^10^Be.^28^ We extracted 15 rock samples from continuous sections measured in the lower member of the Mazamba Formation at GPL-1, GPL-2, GPL-6, and GPL-12 (Figures 3 and 4). To obtain as unaltered and unweathered rocks as possible, samples were taken from freshly excavated trench or section walls. The most fossiliferous and best studied outcrops thus far, GPL-1 and GPL-12, are covered by six and five samples, respectively, that were collected from consecutively younger units present in each section. All sampling positions were documented by total station measurements. Table S1 lists all samples collected for dating together with their paleoenvironmental context interpreted from the sedimentary record.

Besides sampling the sedimentary strata to be dated (“fossil samples”), atmospheric ^10^Be dating requires sampling of sediments from modern environments (“modern samples”) equivalent to those reconstructed from the sedimentary record to determine the initial authigenic ratio N_0_ characteristic of the Gorongosa region.^28^^,^^29^ To obtain these modern sediment samples, of which we analyzed nine in this study (Table S1), a range of environments was sampled, including the banks of three rivers descending from Mount Gorongosa (proximal fluvial settings), the banks of the Pungue and Urema Rivers and the shore of Lake Urema (medial fluvial and lacustrine settings), as well as several localities on the coast, including the Savane River estuary and another estuary northeast of Beira, the shores of which support extensive mangrove swamps and forests (distal coastal, estuarine, and mangrove forest settings).

The authigenic ^10^Be/^9^Be ratios measured for the modern sediment samples (ranging from 70.9 to 281 × 10^−13^, Table S2) are low compared to the range of authigenic ^10^Be/^9^Be ratios of recent surficial continental sediments in general.^30^^,^^31^^,^^32^ Due to the dispersion of the obtained N_0_ values, with a low statistical correlation value, the modern samples were grouped by depositional environments. Then, three scenarios were considered: (1) a direct modern sedimentary/environmental conditions equivalent, (2) a fully estuarine environmental equivalent, and (3) a sedimentary source equivalent. For the first computing (Table 2 part (1)), assuming that the lower Mazamba sediments were deposited in two main paleoenvironments, i.e., fluvio-deltaic and estuarine-lagoonal, we chose modern samples derived from an environmentally equivalent context. For the fossil fluvio-deltaic deposit samples (n = 5) (Be18-Gor-GPL1NE-1, Be18-Gor-GPL1NE-2, Be18-Gor-GPL12–0.1, Be18-Gor-GPL12–1.1, and Be18-Gor-GPL12–4.1), data from the modern sample Be18-Bei-EstRi1-1 were used as the N_0_ reference value to calculate depositional ages of 8.6 ± 0.2 and 14.6 ± 0.3 Ma for the first two samples from the base of GPL-1NE. For samples from the basal and middle sections at GPL-12 (GPL12-0.1, −1.1, and −4.1), deposition ages of 17.1 ± 0.5, 19.5 ± 0.8, and 16.9 ± 0.6 Ma were calculated, respectively. By contrast, the modern estuarine context samples Be18-Bei-SavEst-1 and Be18-Bei-SavFor-1, for which a weighted mean ^10^Be/^9^Be ratio of 0.640 ± 0.034 × 10^−8^ was obtained, were used as N_0_ reference material to calculate deposition ages for the remaining fossil samples (n = 10) that reflect estuarine-lagoonal conditions. Calculated ages for these samples, coming from middle to upper parts of the GPL-1 and GPL-12 sections, range between 6.9 ± 0.2 (Be18-Gor-GPL1NE-6) and 17.8 ± 0.7 Ma (Be18-Gor-GPL1NE-5).

**Table 2:** Computed authigenic ages for the lower member of the Mazamba Formation

Samples	(1) Initial Authigenic ^10^Be/^9^Be∗10^−8^	(1) Initial Authigenic age in Ma	(2) Initial Authigenic ^10^Be/^9^Be∗10^−8^	(2) Initial Authigenic age in Ma	(3) Initial Authigenic ^10^Be/^9^Be∗10^−8^	(3) Initial Authigenic age in Ma
Be18-Gor-GPL1NE-1	13.867 ± 0.521	8.591 ± 0.179	0.640 ± 0.034	7.043 ± 0.190	0.226 ± 0.007	4.958 ± 0.165
Be18-Gor-GPL1NE-2	13.867 ± 0.521	14.568 ± 0.268	0.640 ± 0.034	13.020 ± 0.273	0.226 ± 0.007	10.935 ± 0.252
Be18-Gor-GPL1NE-3	0.640 ± 0.034	8.957 ± 0.199	0.640 ± 0.034	8.957 ± 0.199	0.226 ± 0.007	6.872 ± 0.173
Be18-Gor-GPL1NE-4	0.640 ± 0.034	14.540 ± 0.519	0.640 ± 0.034	14.540 ± 0.519	0.226 ± 0.007	12.455 ± 0.508
Be18-Gor-GPL1NE-5	0.640 ± 0.034	17.779 ± 0.696	0.640 ± 0.034	17.779 ± 0.696	0.226 ± 0.007	15.693 ± 0.687
Be18-Gor-GPL1NE-6	0.640 ± 0.034	6.870 ± 0.227	0.640 ± 0.034	6.870 ± 0.227	0.226 ± 0.007	4.785 ± 0.206
17-Gor-GPL2-5	0.640 ± 0.034	8.940 ± 0.186	0.640 ± 0.034	8.940 ± 0.187	0.226 ± 0.007	6.855 ± 0.158
17-Gor-GPL2-10	0.640 ± 0.034	7.778 ± 0.201	0.640 ± 0.034	7.778 ± 0.201	0.226 ± 0.007	5.692 ± 0.176
17-Gor-GPL6-3	0.640 ± 0.034	10.952 ± 0.225	0.640 ± 0.034	10.952 ± 0.225	0.226 ± 0.007	8.866 ± 0.201
17-Gor-GPL6-8	0.640 ± 0.034	10.761 ± 0.308	0.640 ± 0.034	10.761 ± 0.308	0.226 ± 0.007	8.675 ± 0.291
Be18-Gor-GPL12–0.1	13.867 ± 0.521	17.100 ± 0.450	0.640 ± 0.034	15.552 ± 0.452	0.226 ± 0.007	13.467 ± 0.439
Be18-Gor-GPL12–1.1	13.867 ± 0.521	19.531 ± 0.842	0.640 ± 0.034	17.983 ± 0.843	0.226 ± 0.007	15.898 ± 0.835
Be18-Gor-GPL12–3.1	0.640 ± 0.034	10.887 ± 0.233	0.640 ± 0.034	10.887 ± 0.233	0.226 ± 0.007	8.802 ± 0.209
Be18-Gor-GPL12–4.1	13.867 ± 0.521	16.894 ± 0.570	0.640 ± 0.034	15.346 ± 0.572	0.226 ± 0.007	13.261 ± 0.562
Be18-Gor-GPL12–5.1	0.226 ± 0.007	13.159 ± 0.288	0.640 ± 0.034	13.159 ± 0.288	0.226 ± 0.007	11.073 ± 0.268

(1) Modern environmental equivalent sample used for fossil samples Be18-Gor-GPL1NE-1, Be18-Gor-GPL1NE-2, Be18-Gor-GPL12–0.1, Be18-Gor-GPL12–1.1, and Be18-Gor-GPL12–4.1: Be18-Bei-EstRi1-1; modern environmental equivalent samples used for the other fossil samples: Be18-Bei-SavEst-1 and Be18-Bei-SavFor-1 with a weighted mean ^10^Be/^9^Be ratio of 0.640 ± 0.034 × 10^−8^. (2) Modern estuarine equivalent samples used for all fossil samples: Be18-Bei-SavEst-1 and Be18-Bei-SavFor-1 with a weighted mean ^10^Be/^9^Be ratio of 0.640 ± 0.034 × 10^−8^. (3) Modern source equivalent samples used for all fossil samples: Be18-Gor-Urem-1.1, Be18-Gor-Vun-1.1, and Be18-Gor-VunS1-1.1 with a weighted mean ^10^Be/^9^Be ratio of 0.226 ± 0.007 × 10^−8^.

In the second computing (Table 2 part (2)), assuming the depositional environment for the lower Mazamba Formation was mainly estuarine, only the two modern estuarine context samples (Be18-Bei-SavEst-1 and Be18-Bei-SavFor-1) were considered for age calculations with a mean N_0_ value of 0.64 ± 0.03 × 10^−8^. In this scenario, calculated deposition ages range from 6.9 ± 0.2 (Be18-Gor-GPL1NE-6) to 18.0 ± 0.8 Ma (Be18-Gor-GPL12–1.1) and only the resulting dates for the five fossil fluvio-deltaic samples change with respect to the first computing.

In the third computing (Table 2 part (3)), environmental conditions were largely irrelevant for the choice of modern reference samples. Instead, we chose modern samples for obtaining N_0_ values (mainly for the dissolved ^9^Be input sources) based on sampling localities in the vicinity of the source rocks that the sediments are inferred to be primarily derived from (i.e., Gorongosa Suite granite and gabbro exposed at Mount Gorongosa; Habermann et al., 2019). Matching depositional environments of modern and fossil samples (in this case fluvial) were considered secondarily only in the selection process. The ^10^Be/^9^Be ratios obtained from three modern samples, one from the banks of the Urema River (Be18-Gor-Urem-1.1) and two from the banks of the Vunduzi River (Be18-Gor-Vun-1.1 and Be18-Gor-VunS1-1.1), were used to calculate a weighted mean N_0_ value of 0.226 ± 0.007 × 10^−8^. This weighted mean value was then applied in age calculations to the lower Mazamba samples to be dated. In this approach, resulting ages prove to be slightly younger, ranging between 4.8 ± 0.2 (Be18-Gor-GPL1NE-6) and 15.9 ± 0.8 Ma (Be18-Gor-GPL12–1.1).

Thus, under the three different models, all but two of the samples yield dates within the time frame of the Miocene. The lower sections of GPL-12 yield the oldest dates and indicate that the sediments are of early Miocene age. The four samples from GPL-2 and GPL-6 provide late Miocene ages under the three different models.

### Cosmogenic nuclides - ^26^Al/^10^Be dating

The upper member of the Mazamba Formation has not yielded any fossils yet, and previous geological work indicates it is much younger than the lower member, but no radiometric dates have been previously reported. We applied the ^26^Al/^10^Be burial dating method based on the decay of ^26^Al and ^10^Be cosmogenic nuclides produced *in situ* in quartz (SiO_2_) minerals^33^^,^^34^^,^^35^ to date samples from the upper member and thus provide chronological constraints on the fossiliferous lower member. In general, this technique is applicable for the time frame from 100 ka to ∼6 Ma.^36^ We chose two rock samples collected from two detailed stratigraphic sections in the Mussapassua area in the southeastern corner of GNP where the upper member is well exposed. Under two different models, the samples yielded burial duration dates of 1.316 ± 0.54 and 0.838 ± 0.22 Ma and indicate that at least part of the upper member is of early Pleistocene age (Tables S3 and S4). Further research is needed to evaluate these dates.

### Pedogenic carbonates

**Figure 5:**
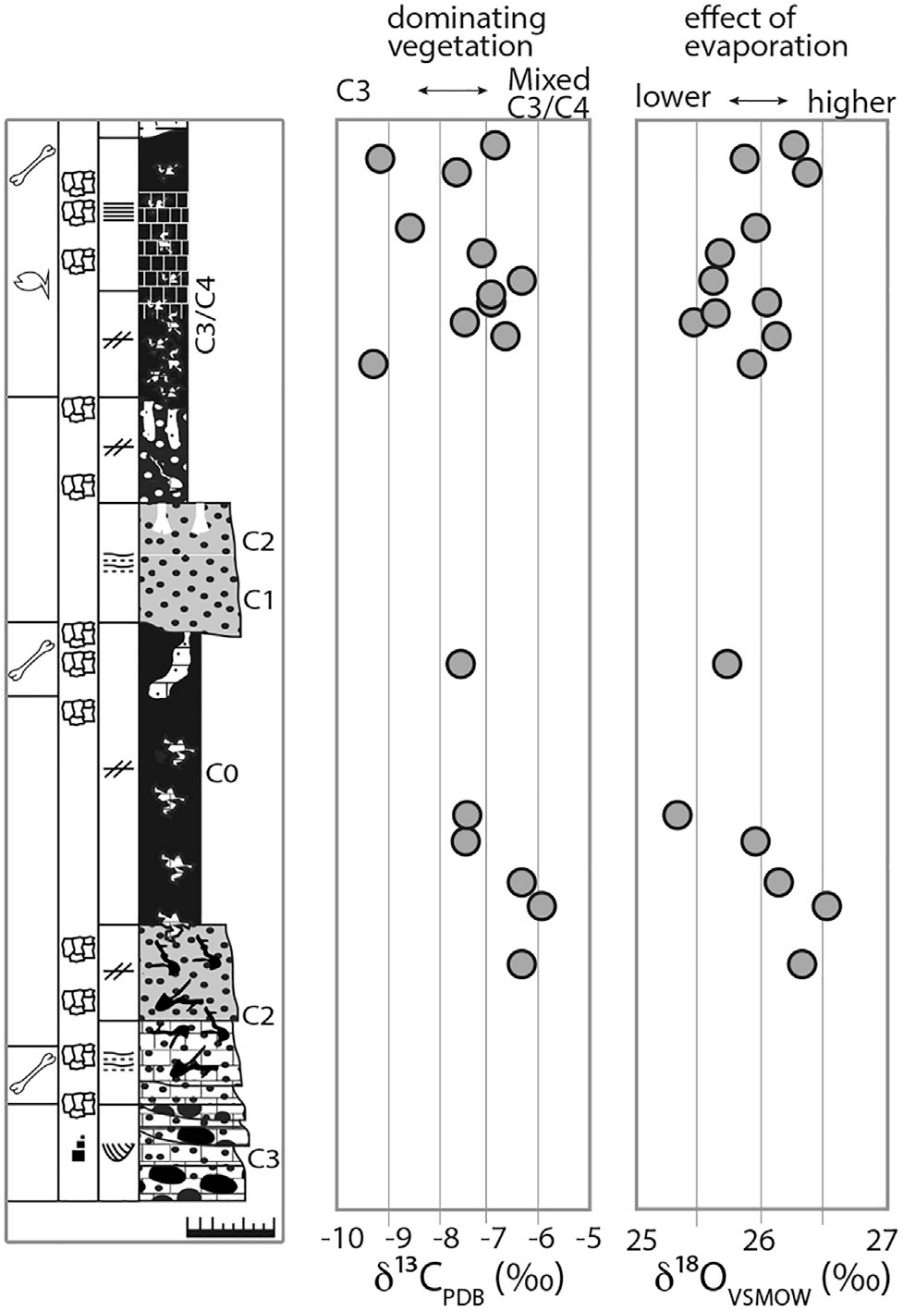
Stable carbon and oxygen isotopes δ^13^C and δ^18^O related to the stratigraphic column of GPL-1NE.

Results of pedogenic stable isotope analysis are listed in Table 3 and shown in Figure 5. Stable carbon isotope ratios of pedogenic carbonates of GPL-1 vary between −9.3% and −5.9% with an average value of −7.3 ± 1.0%, while oxygen isotopes ratios fluctuate from 25.4% to 26.5% with an average of 25.9 ± 0.3%. There is very low correlation between δ^13^C and δ^18^O present (R^2^ = 0.1). Overall stratigraphic trends cannot be detected in either of the two datasets. Carbonate content of the nodules is generally >50% with only one sample having a significantly lower carbonate content (16%), but comparable isotopic values. The average carbonate content is 80 ± 20%.

**Table 3:** Stable carbon and oxygen isotopes

Sample ID	Distance from base [cm]	δ13CVPDB [‰]	δ18OVSMOW [‰]	Weight [μg]	Carbonate content [%]
GLP1-1NE-25	390	−6.8	26.2	112	91
GLP1-1NE-24	385	−9.1	25.8	144	87
GLP1-1NE-23	380	−7.7	26.4	121	89
GLP1-1NE-22	360	−8.6	26.0	129	95
GPL1-1NE-21	350	−7.1	25.7	366	16
GLP1-1NE-19	340	−6.4	25.7	146	96
GLP1-1NE-18	335	−7.0	26.0	135	91
GLP1-1NE-17	330	−7.0	25.6	135	93
GLP1-1NE-16	325	−7.5	25.5	170	89
GLP1-1NE-15	320	−6.7	26.1	139	88
GLP1-1NE-14	310	−9.3	25.9	159	78
GPL1-1NE-13	200	−7.6	25.7	149	83
GPL1-1NE-10	145	−7.4	25.4	123	50
GPL1-1NE-09	135	−7.5	26.0	131	62
GPL1-1NE-08	120	−6.3	26.2	136	79
GPL1-1NE-07	110	−5.9	26.5	131	86
GPL1-1NE-06	90	−6.3	26.3	150	80

Values with sample ID, distance from the base of section GPL-1NE, amount of untreated carbonate powder and carbonate content. For stratigraphic context, see Figure 4.

Carbon isotope values average −7.3 ± 1.0% and never exceed −5.9%. Such low values are typical for C_3_ dominated ecosystems characterized by woodland, bushland, or wooded grassland environments with a mix of C_3_/C_4_ vegetation. Following the vegetation classification of the study by White,^37^ this would indicate average woody cover of at least 50% (for the average δ^13^C value of −7.3%), using the “paleo-shade” proxy.^38^ The oxygen isotopic values of pedogenic carbonates from GPL-1 show fluctuations of only 1.1% toward a relatively persistent climate with no large variation in temperature, source water supply, or effects of evaporation. Without constraints on paleotemperature or ancient soil water oxygen isotopic composition, temporal and geographic variations in fossil soil carbonate δ^18^O values can only be used to identify qualitative changes in climatic patterns, but the relatively low δ^18^O values could indicate a mesic climate with high water supply, which is also supported by the sedimentology, geology, fossil faunal, and floral assemblages of this costal riverine forest/woodland ecosystem.

### Paleobotany

**Figure 6:**
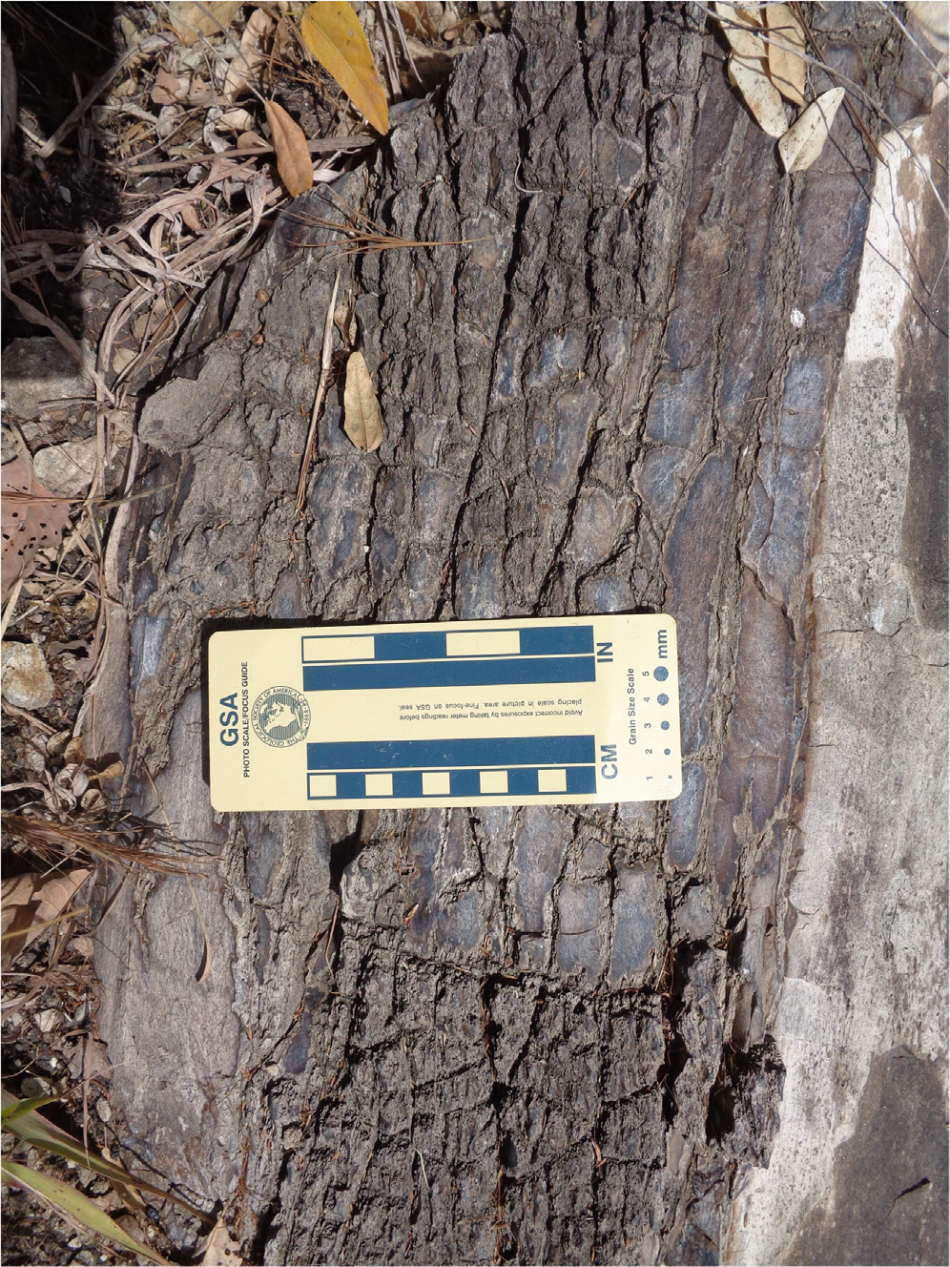
Silicified tree trunk with bark preserved at Menguere Hill

**Figure 7:**
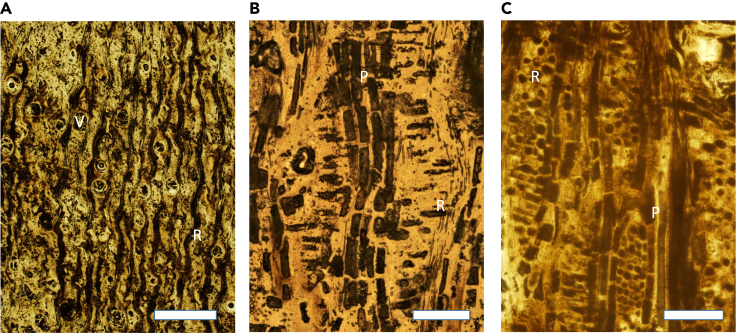
Photomicrographs of thin sections of fossil wood specimen PPP-G-36 from Menguere Hill, *Entandrophragmoxylon* sp. (Meliaceae, African Mahogany) (A) Transverse section showing large mostly solitary vessels, vasicentric to aliform parenchyma, and wide rays with dark contents. (B) Radial longitudinal section with a vertical column of axial parenchyma cells, and horizontal radial parenchyma cells that are procumbent. (C) Tangential longitudinal section with vertical columns of axial parenchyma cells and lens-shaped outline of rays with circular parenchyma cells. Letters: V = vessel; R = ray; P = axial parenchyma. Scale bars: A = 1cm; B, C = 500 μm.

At Menguere Hill, about 3.5 km west of GPL-1, there are large, silicified tree trunks (Figure 6) measuring up to 1.6 m in diameter, as well as scattered fragments of fossil wood. Menguere Hill rises 40 m above the surrounding landscapes and exposes a series of silicified limestone beds. During the 2016–2018 field seasons, we collected 41 specimens of well-preserved fossil wood for microscopic analysis of thin sections and here we present a preliminary taxonomic list and the paleoecological implications of the taxa. Thin sections of the three planes (transverse, radial longitudinal, and tangential longitudinal) of the silicified woods were studied under the microscope and the arrangements of tissues and cell measurements were compared with the anatomy of modern plants in the InsideWood database. For methodological details, see the study by Bamford 2017.^39^ The Gorongosa sample includes the palm *Hyphaene* (Palmae, family Arecaceae), which is widespread in the humid, hot lowlands of tropical Africa. The most abundant taxon in the collection is *Entandrophragmoxylon* (African mahogany, family Meliaceae) (Figure 7). This genus is recognizable by the combination of features: large diameter mostly solitary vessels with simple perforation plates, confluent axial parenchyma and banded parenchyma about 3 cells wide, 2-6-seriate rays with procumbent body cells, and one row of marginal upright cells, often containing crystals, and the inter-vessel pitting is small. The modern genus *Entandrophragma* is restricted to tropical Africa, and some species can reach up to 60 m in height. We have previously reported the presence of *Terminalioxylon* (family Combretaceae),^18^ a genus that is most diverse in bushveld and savannas, and includes some mangrove species. There are also samples of *Ziziphus* (family Rhamnaceae), which is common along watercourses, and *Zanha* (family Sapindaceae), found in open woodland to dense ravines and riverine forests.^40^^,^^41^^,^^42^ A further observation to note is that cross sections of the wood vessels indicate mesophytic trees that cannot tolerate water stress. We interpret the Menguere Hill succession as a correlative inland equivalent to the estuarine fossil sites farther to the east based on similar elevations.^18^

### Systematic paleontology

Here, we describe several specimens from the lower Mazamba Formation found during the 2016–2019 field seasons. All fossil specimens are listed in the Paleo-Gorongosa Database, where each entry provides specimen number, locality, GPS coordinates, stratigraphic position, taxonomic attribution, and skeletal elements represented. Each specimen has the prefix PPG followed by the year of discovery, as in PPG2017-P-121. Following the prefix and year of discovery, the letter P refers to Paleontological collection (rather than archaeological or osteological collections). Specimens were numbered sequentially as they were retrieved in the field each year. All specimens are housed in the Paleontology Laboratory in Chitengo, Gorongosa National Park.

Class Chondrichthyes Huxley, 1880
Subclass Elasmobranchii Bonaparte, 1838
Order Carcharhiniformes Compagno, 1977
Family Carcharhinidae Jordan & Evermann, 1896
Genus *Galeocerdo* Müller & Henle, 1837
Referred specimens: PPG2017-P-121 from GPL-1, PPG2018-P-224 from GPL-1, PPG2019-P-126, 129, 176 from GPL-12
*Galeocerdo aduncus* Agassiz, 1843
Referred specimen: PPG2019-P-127 from GPL-12

**Figure 8:**
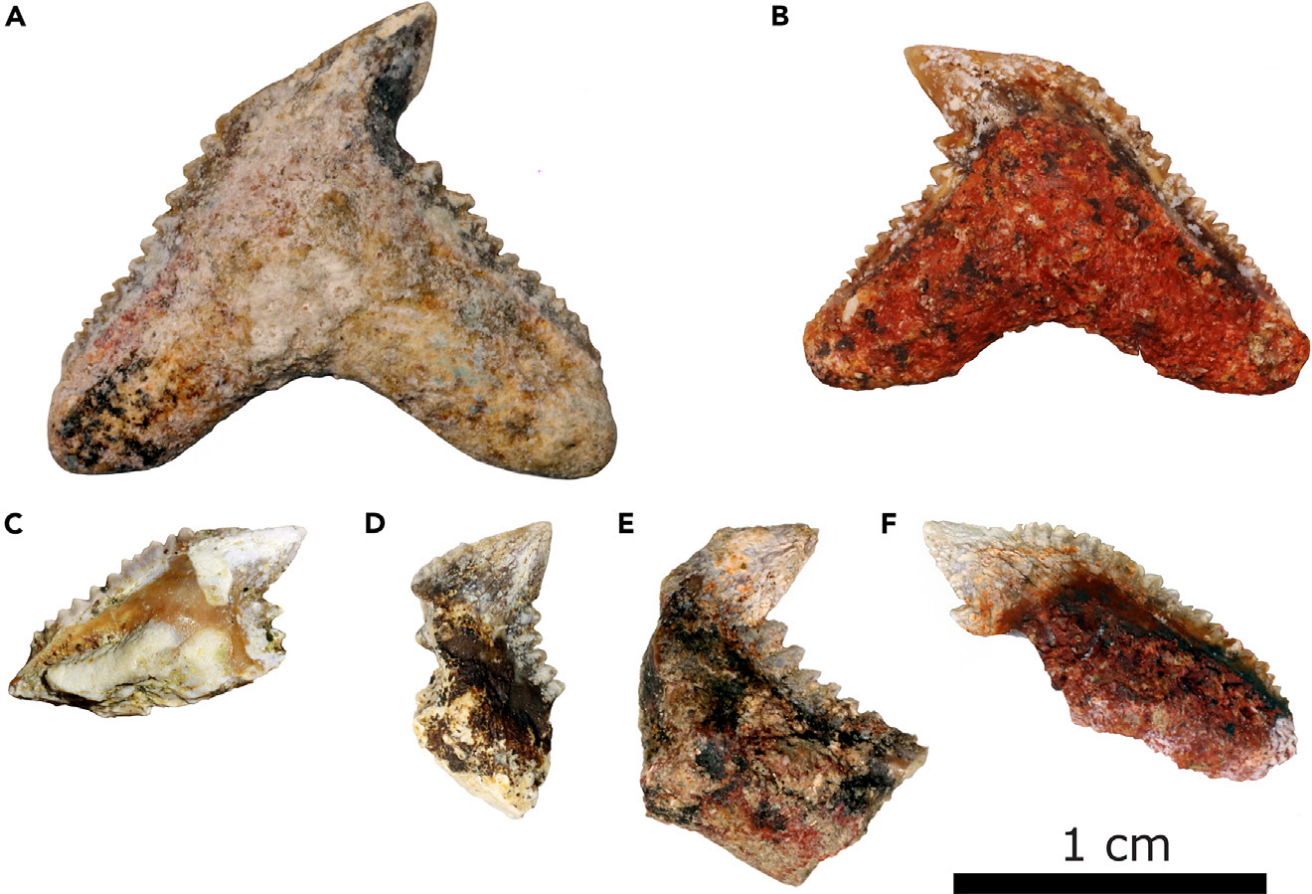
Gorongosa fossil sharks, all in the genus *Galeocerdo*, tiger sharks (A) PPG2019-P-129. (B) PPG2019-P-127. (C) PPG2018-P-224. (D) PPG2019-P-176. (E) PPG2017-P-121. (F) PPG2019-P-126.

Six specimens of shark teeth were recovered from the Gorongosa sedimentary sequence during the 2016–2019 field seasons. Four of these are fragmentary teeth from GPL-1 (PPG2017-P-121, PPG2018-P-224) and GPL-12 (PPG2019-P-126, PPG2019-P-127), and two are complete crowns and roots from GPL-12 (PPG2019-P-127, PPG2019-P-129) (Figure 8). For shark teeth, we use the terminology of Türtscher et al.^43^ The following descriptions and analyses are based on the two complete teeth. One of these teeth (PPG2019-P-129), however, has some weathering on the apex that removed part of the distal cutting edge. The apex of the Gorongosa teeth is dominated by a primary cusp that leans distally. Serrations are present in the mesial cutting edge and the distal heel, but only lightly developed or absent along the apex. The mesial cutting edge has more than a dozen primary serrations that decrease in size away from the apex. The heel is relatively straight and with primary serrations decreasing in size distally. The serrations are simple (not compound), with only primary serrations visible (no secondary serrations). The outline of the mesial cutting edge has a distinct break between the apex and the rest of the serrated mesial cutting edge with two lines meeting at an obtuse angle (140° in PPG2019-P-127 and 155° in PPG2018-P-129). The length of the apex is one-third or less of the length of the rest of the mesial cutting edge. The mesiodistal length of the tooth exceeds its height. The root is relatively thick, bilobate, and well-arched, with the slightly asymmetrical lobes forming an obtuse angle. The six specimens differ in coloration, weathering, and preservation, and appear to represent distinct individuals deriving from two localities separated by ∼700 m. In overall characteristics, the shark teeth have the cockscomb appearance typical of the genus *Galeocerdo*, tiger sharks.

To assess the taxonomic affinities of the Gorongosa shark specimens, we carried out a series of 2D morphometric analyses of the two complete specimens. We compiled a set of fossil shark photographs from the existing literature to obtain a suitable comparative sample of 600 specimens (Table S5). From this comparative sample, we used three datasets including: 1) all 600 specimens from four different genera (*Galeocerdo*, *Physogaleus*, *Carcharhinus*, and *Hemipristis*), 2) a subset of 547 specimens from species of *Galeocerdo* and *Physogaleus*, and 3) a subset including 436 specimens from different species of the genus *Galeocerdo*. We carried out principal component analyses (PCA) of these datasets followed by multi-group linear discriminant analyses (LDA) to classify the Gorongosa specimens into taxonomic categories (STAR Methods).

**Figure 9:**
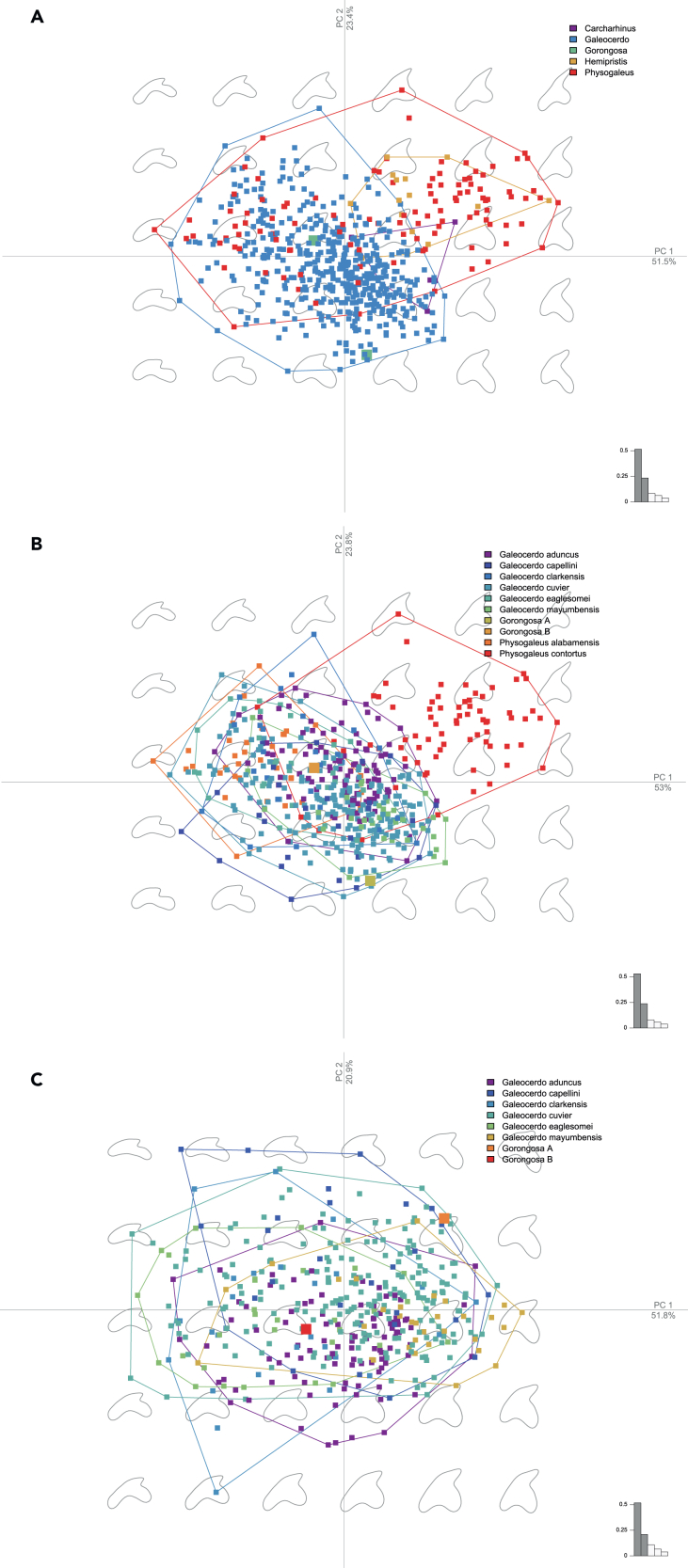
Fossil shark principal component analysis (A) PCA of 600 Miocene shark teeth from the genera *Carcharhinus*, *Galeocerdo*, *Hemipristis*, and *Physogaleus*, and including the two Gorongosa complete crowns. (B) PCA of 547 Miocene shark teeth of the species *Galeocerdo* sp., and *Physogaleus* sp., and the Gorongosa specimens. (C) PCA of shark teeth including the species *G. aduncus*, *G. capellini*, *G. clarkensis*, *G. cuvier*, *G. eaglesomei*, and *G. mayumbensis*, with the Gorongosa specimens.

The first PCA considering four genera of sharks shows that both Gorongosa specimens are located within the convex hulls of *Galeocerdo* (Figure 9A). In the second PCA, considering eight species of *Galeocerdo* and *Physogaleus*, Gorongosa B (PPG2017-P-127) is located near the center of the *Galeocerdo aduncus* convex hull, while Gorongosa A is in a marginal position near the edges of *G. cuvier* and *G. capellini* (Figure 9B). In the third PCA, which considers only species of *Galeocerdo*, Gorongosa B is again near the center of the *G. aduncus* convex hull, while Gorongosa A is near the edges of *G. cuvier* and *G. capellini* (Figure 9C). The three LDA models using the principal components (PCs) that accounted for 90% of the variance of the sample clearly distinguish among the taxonomic categories, displaying good performances with satisfactory classification results after cross-validation (Table S6). When using the obtained discriminant functions to classify the Gorongosa fossil sharks into these taxonomic categories (as a way of assessing morphological affinities), they were robustly classified within the genus *Galeocerdo*. When classifying the fossils using the species categories, Gorongosa A was classified within *Galeocerdo cuvier*, while Gorongosa B was strongly categorized within *Galeocerdo aduncus*. Gorongosa specimen PPG2019-P-127 shares with *A. aduncus* a lack of secondary serrations on the mesial cutting edge and slightly asymmetric roots.

The size and morphology of the fragmentary teeth in the Gorongosa collection is consistent with those of the complete crowns, and we attribute all six specimens to the same genus. *Galeocerdo* upper and lower teeth are very similar, but they increase in breadth relative to height posteriorly. The teeth of juvenile tiger sharks have fewer serrations than those of adults.^43^ The Gorongosa fossil teeth are functionally similar to those of the extant tiger shark, and we may infer similar function in piercing large prey.

Batoidea Compagno, 1973
Order Myliobatiformes Compagno, 1973
Referred specimen: PPG2018-P-257 from GPL-1

A single fragment of batoid symphyseal teeth was found at GPL-1. This indicates that at least two taxa of cartilaginous fishes occur in the Gorongosa fossil record, one species of shark and one species of ray. Most batoid species live in tropical and subtropical coastal waters, and some can occur in estuaries.

Order Testudines Batsch, 1788
Referred specimens: PPG2016-P-12, 13, 14, 27, 55, PPG2017-P-42, 44, 87, 95, PPG2018-P-10, 201, 203, 206, 217, 233, 234, 235, 270, 271
Family Testudinidae Batsch, 1788
Referred specimen: PPG2016-P-9

**Figure 10:**
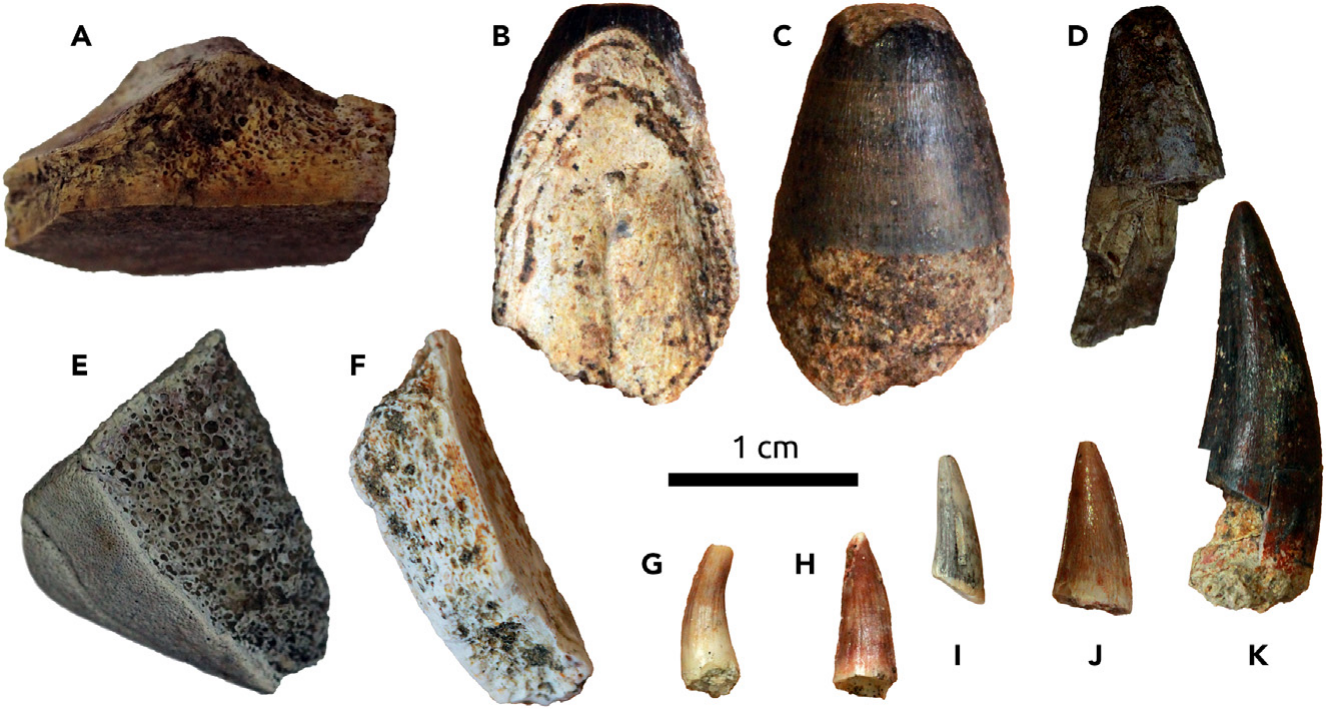
Some fossil reptiles from Gorongosa (A, E, and F) Testudines. (B–D and G–K) Crocodylia.

There are 20 specimens of turtles and tortoises in the Gorongosa fossil collections, which include fragments of carapace and plastron. One of the first specimens to be recovered in the field was PPG2016-P-9, a plastron fragment consistent in thickness and morphology with terrestrial tortoises (family Testudinidae) (Figure 10A), which have been present in Africa since the late Eocene.^44^^,^^45^ Most specimens are fragmentary but further analyses will aim to refine the taxonomic attributions.

Order Crocodylia Gmelin, 1789
Family Crocodylidae Cuvier, 1807
Crocodylidae indet.
Referred specimens: PPG2016-P-10, 23, PPGG2017-P-43, 49, 73, 80, 89, PPG2018-P-100, 161, 162, 222, 223, 241, 252, 264, PPG2019-P-116, 117, 128

There are 18 teeth and tooth fragments attributed to Crocodylidae. Their abundance attests to relatively stable bodies of water in the region. Tooth crown morphologies are consistent with size and shape heterodonty in brevirostrine taxa (Figure 10).^46^^,^^47^ Although represented by small sample sizes, maximum tooth crown lengths indicate body sizes similar to comparatively small-bodied crocodylids from the Paleogene and early to middle Miocene of North African and sub-Saharan formations,^48^^,^^49^^,^^50^ as opposed to the gigantic late Miocene-Pleistocene taxa from East Africa.^51^^,^^52^ A single broken, poorly preserved tooth is elongate and slightly recurved distally, similar to the condition in longirostrine, piscivorous tomistomine, and gavialoid taxa, suggesting the presence of at least two crocodylid taxa in the lower member of the Mazamba Formation.

Mammalia Linnaeus, 1758
Afrotheria Stanhope et al., 1998
Order Hyracoidea Huxley, 1869
Family Saghatheriidae Andrews, 1906
gen. et sp. nov.
Referred specimens: PPG2018-P-1, 2

**Figure 11:**
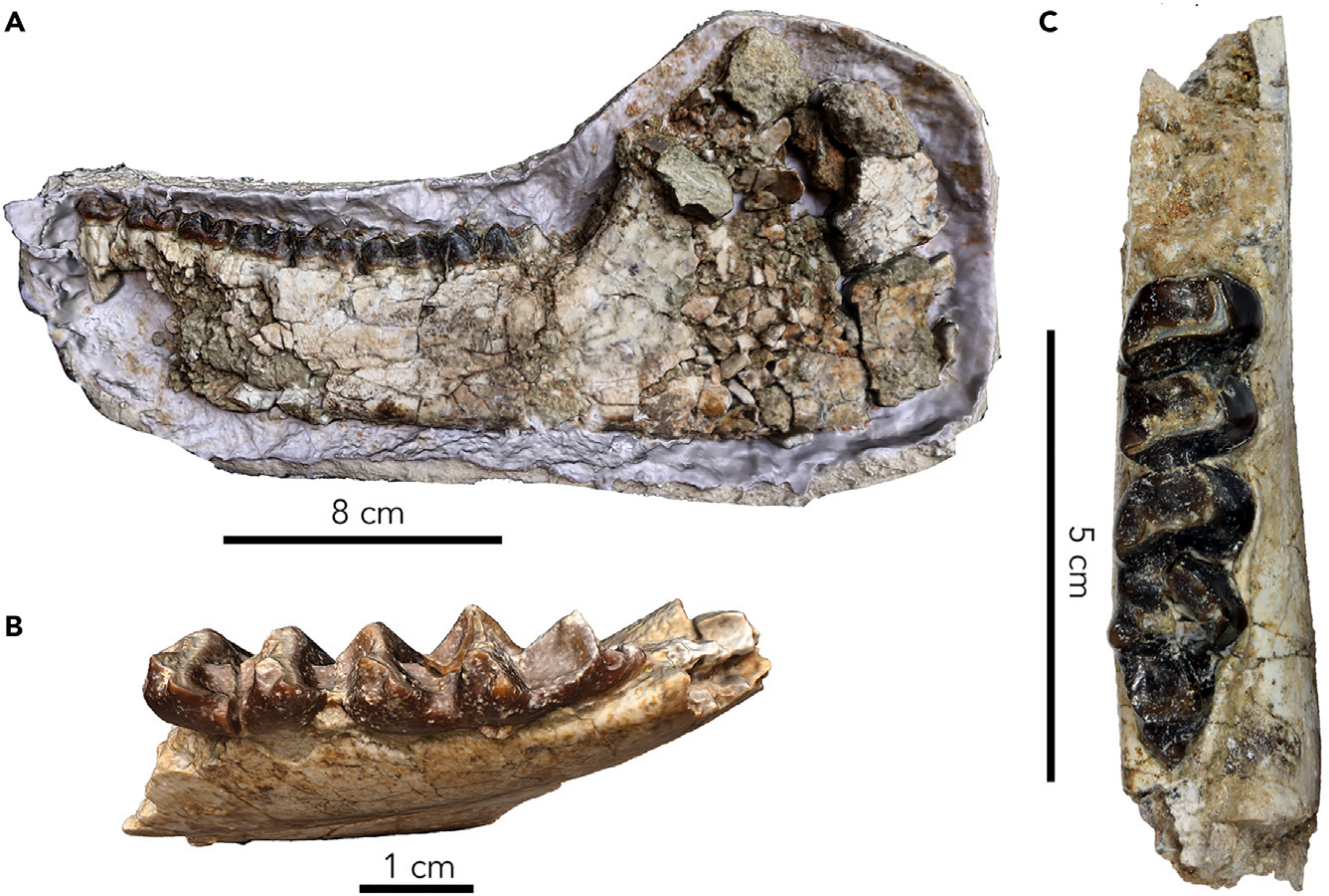
Fossil hyracoids (A) Hyracoid left mandible PPG2018-P-1. (B) Hyracoid right mandibular fragment, PPG2018-P-2. (C) PPG2018-P-2 in occlusal view.

Hyraxes (order Hyracoidea) belong to the Afrotheria, a clade of mammals with deep evolutionary roots in Africa. The Gorongosa sample includes an individual with left and right mandibular fragments (Figure 11) excavated *in situ* from Facies 2 at GPL-12. The hyracoid mandibles represent some of the oldest mammals found so far in the Gorongosa sequence (early Miocene based on the atmospheric ^10^Be dates). The left hemimandible (PPG2018-P-1) has the complete premolar-molar dentition, from p1 to m3, but the specimen is extremely fragile, so it remains in its plaster jacket for protection and only the buccal and occlusal aspects are visible. The right mandible fragment (PPG2018-P-2) has a set of molars m2-m3 and three detached premolars (p2, p3, and p4). Tooth measurements are given in Table 4. The mandibular body, as seen on the left side, shows a slight depression on the buccal side below the level of m1-m2. The cheek teeth increase monotonically in mesio-distal length from p1 (12.81 mm) to m3 (31.01 mm). The teeth are brachydont, and the molars are bilophodont with well-developed transverse crests. The posterior premolars, p3-p4, are molarized. In the molars, the protoconid is large and gives rise to the protocristid that extends to the metaconid and forms the mesial loph at the back of the trigonid. The paraconid is reduced and the metaconid is the tallest cusp. The hypoconid gives rise to a marked hypocristid that extends to the entoconid and forms the distal loph at the back of the talonid. The molars also contain lingual spurs in the metaconid and entoconid that extend linguo-distally. The m3 has a well-developed hypoconulid and a third loph joins the hypoconulid with the endoconulid. The distal cingulum forms a distinct posterior cusplet in the m3, a feature that seems to be rare in hyracoids, but is present in *Thyrohyrax kenyaensis* (KNM-NW 58339) from the early Miocene of Nakwai,^53^ and in *Regubahyrax selleyi* (M 82369) from the early Miocene of Libya,^54^ both allied to Saghatheriidae. A continuous cingulum occurs along the mesial, buccal, and distal parts of the molars. The well-developed transverse crests and the low-crowned molars of the Gorongosa specimens most likely indicate a folivorous diet based on soft leaves.

**Table 4:** Measurements of hyracoid teeth in mm

PPG2018-P-1	Side	mesio-distal	bucco-lingual
p1	Lt	12.81	n.a.
p2	Lt	14.64	n.a.
p3	Lt	14.96	n.a.
p4	Lt	15.72	n.a.
m1	Lt	17.59	n.a.
m2	Lt	19.61	n.a.
m3	Lt	31.01	n.a.

**PPG2018-P-2**			

p2	Rt	14.48	8.93
p3	Rt	16.14	10.26
p4	Rt	16.63	12.60
m2	Rt	20.77	15.31
m3	Rt	32.40	14.46

**Figure 12:**
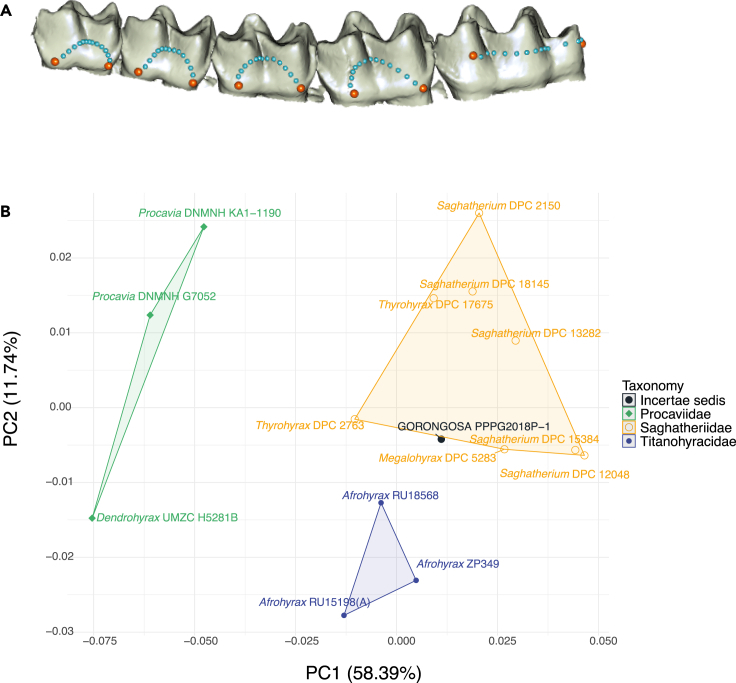
Shape analysis of hyracoid p3-m3 (A) *Thyrohyrax* specimen (DPC 2763) showing the landmarks (orange spheres) and semi-landmarks (light blue spheres) used in this study. This specimen was selected to display the 3D coordinates as it corresponds to the specimen closest to the multivariate mean in this analysis. (B) Principal component analysis (PCA) of the dental shape variables (only the two first PCs are shown).

To compare the Gorongosa mandibles with those from other sites, we carried out a PCA of dental shape variables. For the left hemimandible (PPG2018-P-1), we used five curves with 15 landmarks each from the buccal side (given that the lingual side is obscured by the plaster jacket) to produce dental row outlines from p3 to m3 (Figure 12A). These landmarks were collected using the software Landmark Editor 3.6.^55^ We chose the p3-m3 sequence (excluding p1-p2) to maximize the number of comparative specimens that could be used. We obtained similar outlines from the 3D models of 14 hyracoids. Three of these comparative specimens are housed at the National Museums of Kenya (NMK) and were digitized using photogrammetry following the protocol described by Bucchi and colleagues.^56^ Eleven additional comparative specimens were downloaded from Morphosource https://www.morphosource.org/ ^57^ (Table S7). This comparative sample included the genera *Saghatherium*, *Thyrohyrax*, *Megalohyrax*, and *Afrohyrax* and the modern genera *Dendrohyrax* and *Procavia*. The first and last landmarks from each one of the five curves were treated as fixed (i.e., 10 fixed landmarks), whereas all the rest of them (i.e., 65 landmarks) were considered as semi-landmarks. This PCA shows that the Gorongosa mandible is closer to specimens of Saghatheriidae (*Saghatherium*, *Thyrohyrax*, and *Megalohyrax*) than to Titanohyracidae (*Afrohyrax*) or modern Procaviidae (*Dendrohyrax* and *Procavia*) (Figure 12B) when considering the two first PCs that account for ∼70% of the variance of the sample.

**Figure 13:**
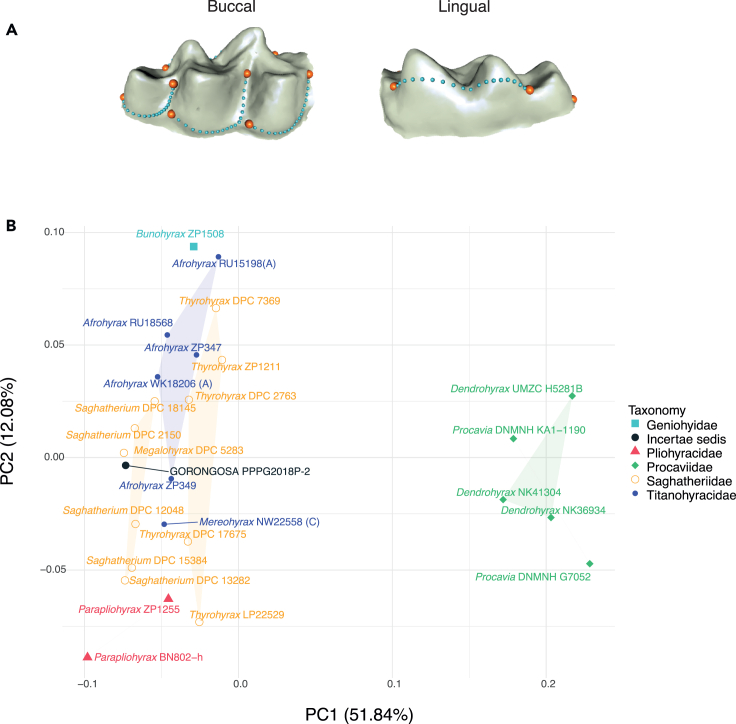
Shape analysis of hyracoid m3 (A) *Afrohyrax* specimen (ZP349) showing the landmarks (orange spheres) and semi-landmarks (light blue spheres) used in this study. This specimen was selected to display the 3D coordinates as it corresponds to the specimen closest to the multivariate mean in this analysis. (B) Principal component analysis (PCA) of the m3 shape variables (only the two first PCs are shown).

In another analysis using only the m3 from mandible PPG2018-P-2, we used four curves with 10 landmarks each (Figure 13A). This dataset was then compared with the 3D models of 25 hyracoids. Thirteen of these specimens are also housed at the NMK and that were digitized using photogrammetry with the same protocol that was described previously, while the rest of the sample was obtained from MorphoSource https://www.morphosource.org/ (Table S8). The comparative sample derives from five families of Hyracoidea: Geniohyidae (*Bunohyrax*), Saghatheriidae (Saghatherium, *Thyrohyrax*, *Megalohyrax*), Titanohyracidae (*Afrohyrax*, *Mereohyrax*), Pliohyracidae (*Parapliohyrax*), and Procaviidae (*Dendrohyrax* and *Procavia*). This dataset was also subjected to a General Procrustes analysis to obtain shape variables. The first and last landmarks from each one of the four curves were treated as fixed (i.e., eight fixed landmarks), while the remaining 3D coordinates (i.e., 32 landmarks) were considered as semi-landmark and were slid by using Procrustes distance minimization as criterion. The obtained shape residuals were then used to carry out a PCA. This PCA shows that the Gorongosa m3 is closer to specimens of Saghatheriidae than to those of other families (Figure 13B) when considering the first two PCs that account for ∼64% of the variance of the sample.

The Gorongosa species is a large hyracoid (body mass ∼124–153 kg) presenting the following autapomorphies: a present p4 premetacristid and an m1 talonid that is shorter than the trigonid. It differs from most hyracoids, while sharing with *Prohyrax hendeyi* and *Procavia capensis*, by having a p1 entoconid that is present but smaller than the hypoconid. It also differs from most other hyracoids, while sharing with *Prohyrax hendeyi*, in having a p2 metaconid that is small relative to the protoconid. It also shares with *Procavia capensis* a p2 entoconid that is well-developed and approximately equal in size with respect to the hypoconid. It differs from *Thyrohyrax* species in exhibiting lower molar buccal cingulids that are present and continuous, as well as exhibiting a trenchant crest connecting hypoconids and hypoconulids on m1-2. It differs from *Prohyrax hendeyi* and *Procavia capensis* in that the position of the metaconid relative to the protoconid on p4 is situated transversally rather than distally, and that the cristid obliqua meets the hypoconid at a sharp angle on m1-m2. It also differs from them and from *Thyrohyrax meyeri* and *Thyrohyrax domorictus* (its closest relatives based on our phylogenetic results) in that relative width of the p4 talonid is approximately equal in width to the trigonid, as well as in that the length of p4 is ∼80%–89% relative to m1. Gorongosa also differs from the rest of hyracoids, excepting a few *Titanohyrax* species, in showing an m1 area ∼200–250 mm^2^. The Gorongosa hyracoids also differ from all *Thyrohyrax* species, *Procavia capensis*, and *Prohyrax hendeyi* in that molar crowns are buccally inflated, and the hypoconids and protoconids are centralized relative to the crown base. It also differs from them in that the orientation of the cristid obliqua on m1 and m2 terminates between the metaconid and protoconid.

**Figure 14:**
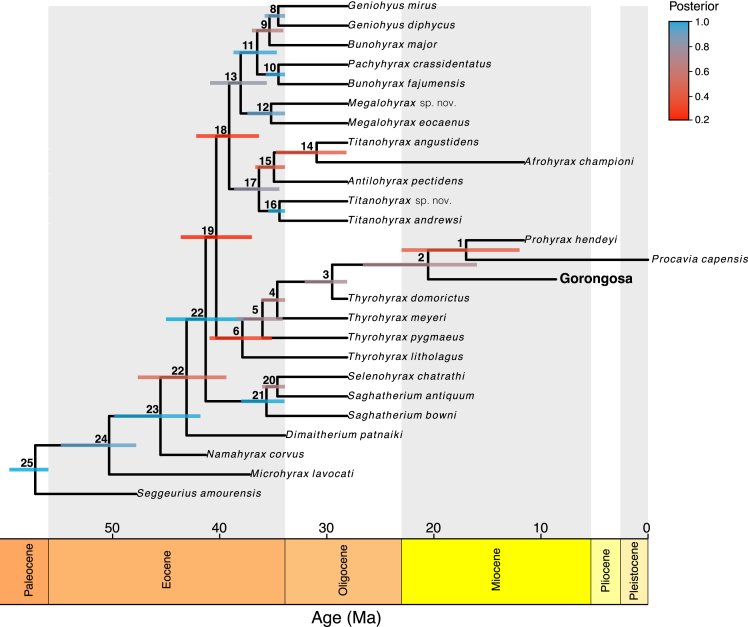
Hyracoid phylogeny Maximum credibility (MCC) tree summarizing 75,000 hyracoid phylogenies obtained from a Bayesian phylogenetic analysis. The length of the bars on the MCC tree corresponds to the temporal 95% highest posterior density interval (HPD), while the color represents posterior support. Numbers on the phylogeny correspond to node numbers in Table S9.

To infer the evolutionary relationships of the Gorongosa specimens, we carried out a Bayesian phylogenetic analysis of hyracoid species (Figure 14), combining morphological and stratigraphic range data from the fossil record using RevBayes v.1.1.0.^58^ The morphological data came from Cooper et al.^59^ and comprised a supermatrix of 403 morphological characters from where we extracted all the hyracoid species present.^59^ We collected all mandibular characters available in the Gorongosa hyracoid materials and added this information to the hyracoid morphological matrix. The stratigraphic ranges are the first and last occurrences observed for a single species in the fossil record and were obtained from the Paleobiology Database at https://paleobiodb.org (Table S9).

After discarding a 25% burn-in, we computed a maximum credibility (MCC) tree as a way of summarizing our posterior tree sample. Overall, our hyracoid tree is mostly well resolved showing high posterior support with ∼72% of the nodes displaying posterior values larger than 0.5 (Figure 14). The topology of our MCC tree is highly similar to the topology for the hyracoid clade obtained by Cooper et al.^59^ using parsimony but better resolved. In general, the polytomies obtained by Cooper et al.^59^ correspond to nodes displaying the lowest support in our phylogeny. The only topological differences between both trees occur in the clade comprising *Afrohyrax championi*, *Antilohyrax pectidens*, and *Titanohyrax angustidens* (a clade with high topological uncertainty), as well as—of course—the inclusion of the Gorongosa materials. The Gorongosa hyracoids correspond in our phylogeny to the sister clade of the most recent hyracoids analyzed by us (i.e., *Procavia capensis* and *Prohyrax hendeyi*). Although not the main focus of our present study, our analyses also provide divergence time estimates, including a speciation time for the Gorongosa hyracoids at around ∼21.3 Ma (Table S9).

## Discussion

The new fossil sites from Gorongosa National Park open an entirely new vista on a region of Africa that, until now, had remained paleontologically unknown (Figures 1 and 2). No other sites along the East African Rift System yield the combination of fossil woods (e.g., African mahogany), marine invertebrates (crabs, gastropods, bivalves), marine vertebrates (sharks and rays), reptiles (crocodiles, tortoises), and terrestrial mammals (e.g., hyracoids). The geological, sedimentological, paleobotanical, geochemical, and paleontological evidence indicates that the Gorongosa fossil sites formed in coastal settings, even though today these sites are ∼95 km from the modern coast and at ∼100–120 m above sea level (Figures 3 and 4).

The new fossils derive from multiple sedimentary beds across ten paleontological localities in the lower member of the Mazamba Formation. Previous geological work assigned this sedimentary sequence broadly to the Miocene,^22^^,^^23^^,^^26^ but no radiometric dates had been obtained prior to our work. Here, we have presented the first atmospheric beryllium dates for the Mazamba Formation (Table 4). Atmospheric beryllium samples from the lower member range in age from the early to the late Miocene and confirm the broad placement of this part of the sequence in the Miocene. Two samples from the lowermost sections of GPL-12 provide an early Miocene age for the fluvio-deltaic sediments from which some key fossils derive. Atmospheric beryllium samples from GPL-2, which we expect to be younger based on our tentative correlations (Figures 3 and 4), indicate a late Miocene age for those sediments (Table 4).

The sedimentological, isotopic, paleobotanical, and paleontological evidence presented here indicates that the fossil sites formed in coastal woodlands or estuarine conditions. At GPL-1, for example, paleosol carbon and oxygen isotopes indicate the prevalence of C_3_ vegetation (trees, shrubs) with some areas of grassland under mesic climate with a high supply of fresh water (Figure 5). This view is supported by the fossil wood (Figure 6), whose most abundant component is *Entandrophragmoxylon* (African mahogany) (Figure 7), a genus that typically grows in areas of high rainfall. There were also palm trees of the genus *Hyphaene*, which are widespread in the humid, hot lowlands with high water tables of tropical Africa today. Other trees in the ancient Gorongosa landscapes include *Terminalioxylon*, which includes some mangrove species, *Ziziphus*, which is common along the edges of watercourses, and *Zanha*, a genus associated with open woodland to dense ravines and riverine forests. Cross sections of the fossil wood vessels indicate the presence of mesophytic trees that cannot tolerate water stress. Thus, these different lines of evidence indicate that terrestrial environments near the coast were consistently warm and wooded, with a prevalence of C_3_ vegetation under mesic conditions.

The rivers descending from the west meandered on a low-gradient coastal plain, where they gave rise to estuaries near shallow marine environments.^18^ Sharks of the genus *Galeocerdo* (Figures 8 and 9) were top predators in these estuaries and nearshore environments. Specimens of *Galeocerdo* are known from the Eocene to the present,^43^ while the species *G. aduncus*, present in the Gorongosa sample, has a temporal range from the Oligocene to the late Miocene.^43^^,^^60^ The genus was widely distributed in the tropical and temperate seas of the Miocene, with specimens found in Madagascar,^61^ North Africa,^62^^,^^63^ Oceania,^64^ Eurasia,^65^^,^^66^ and the Americas.^67^^,^^68^ Modern *Galeocerdo* ranges from pelagic waters to nearshore environments in tropical and subtropical marine ecosystems, often occurring in river estuaries. Tiger sharks are top predators, with a diet of cephalopods, fish, turtles, and other vertebrates.^69^ Like the modern tiger sharks, *Galeocerdo* in the past was a highly mobile apex predator that played a major role in structuring coastal ecosystems.^70^ The presence of these shark fossils in the Miocene of GNP is consistent with our interpretation of estuarine depositional environments.

The fossils analyzed here include Hyracoidea, an order of mammals that belongs to the Afrotheria, a clade with deep evolutionary roots in Africa. There are five species of modern hyraxes, all in the family Procaviidae, but in the past there were at least four additional families: Geniohyidae, Saghatheriidae, Titanohyracidae, and Pliohyracidae. Hyracoids in the Paleogene of Africa were abundant and diverse, both taxonomically and functionally, but declined in overall diversity during the late Miocene.^71^ The chewing teeth of the Gorongosa hyracoid are brachydont and bilophodont, very likely for a diet of relatively soft leaves. The Gorongosa hyracoids represent a very large species (∼124–153 kg) with affinities to taxa in the family Saghatheriidae, but different from currently known species (Figures 11, 12, 13, and 14). The family Saghatheriidae includes the genera *Microhyrax*, *Saghatherium*, *Selenohyrax*, *Thyrohyrax*, *Megalohyrax*, and *Regubahyrax* spanning from the Eocene to the early Miocene. Specimens of *Regubahyrax* from the early Miocene of Libya document the latest known occurrence of saghatheriids.^54^ The lower molars of *Regubahyrax* have well-developed cristids and spurs, but the spurs are not as developed in the Gorongosa hyracoid. Our phylogenetic analysis (Figure 14) confirms this initial assessment as the Gorongosa specimen, with *Prohyrax* and *Procavia* as a sister clade of all the analyzed *Thyrohyrax* species. However, the Gorongosa specimen likely represents a new species.

The fossils documented here represent the first descriptions of a substantial fossil record that is just emerging. The Gorongosa paleontological record opens up the possibility of testing important hypotheses about the role of the eastern coastal forests in shaping the evolution of African mammals.^9^^,^^10^ As the fossil record from Gorongosa is further described and analyzed, it will yield a powerful database spanning different intervals of the Miocene, which will then be compared to other sites on the continent. Thus, we will be able to assess the effects of the northeast-southwest arid corridor in promoting the geographic isolation and evolutionary trajectories of coastal forest plant and animal communities in the past.^72^ The Gorongosa fossil record points to the persistence of woodlands and wooded grasslands along the southeastern coast of Africa during the Miocene, but further work is needed to assess the taxonomic affinities of the Gorongosa mammals with contemporaneous faunas elsewhere in Africa.

### Conclusions

After four field seasons (2016–2019), extensive surveys, and new approaches in the search of paleontological sites,^17^ the Paleo-Primate Project Gorongosa has 1) documented ten new paleontological localities, 2) established a preliminary stratigraphic and sedimentological framework for the fossil sites, 3) provided the first radiometric age determinations for the Mazamba Formation, 4) provided the first reconstructions of past vegetation in the region combining pedogenic carbonates and fossil wood, and 5) described the first fossil teeth from the southern East African Rift System. The Gorongosa fossil record includes new species of mammals, and a unique combination of specimens straddling the terrestrial/marine biomes, with paleoenvironmental evidence for persistent woodlands and forests on the coastal margins of southeastern Africa during the Miocene.

### Limitations of the study

In the main text, we have presented the broad geological background of the Gorongosa paleontological sites, with the Mazamba Formation consisting of a lower member and an upper member. The lower member is sometimes referred to as the “purple sands,” while the upper member was originally called the Inhaminga beds.^22^^,^^23^ This terminology is followed by most subsequent researchers.^18^^,^^25^^,^^26^^,^^73^ However, some of the subsequent published descriptions of sequences above the Cheringoma Formation have offered conflicting and inconsistent terminology.^74^^,^^75^^,^^76^ These large-scale compendia and descriptions are inconsistent with earlier terminology and contain errors that do not make stratigraphic sense (e.g., late Miocene sediments underlying Eocene sediments). Given these inconsistencies, we follow the terminology of Real,^23^ Flores,^22^ Tinley,^26^ Laumanns,^25^ Arvidsson,^73^ and Habermann et al.^18^ in referring to the post-Cheringoma Formation sequence as the Mazamba Formation with a lower and an upper member. However, it is clear that further geological and stratigraphic work is needed to be focused on the Cenozoic sequences of the Cheringoma Plateau.

While authigenic ^10^Be/^9^Be cosmogenic nuclide dating has the advantage that it can be used to date a wide range of rock types, and thus is not limited to volcanic ashes, one of its weaknesses is that it relies on the reconstruction of the depositional environment and the determination of the initial authigenic ^10^Be concentration in order to obtain accurate dates. This can pose a significant challenge if the depositional environment and initial concentration are not well constrained or if these are in contexts difficult to determine. Further applications of different dating techniques, such as uranium series, paleomagnetism, and biochronology, are underway and will further refine the chronology of the sites.

Although the comparative sample used for the analysis of fossil sharks is large and represents a wide range of time intervals, the comparative sample of hyracoids will need to be expanded to include additional specimens of Neogene age from across Africa and other regions.

## STAR★Methods

### Key resources table

**Table:** 

REAGENT or RESOURCE	SOURCE	IDENTIFIER
**Deposited data**		

Hyracoid 3D models (mandibles)	Morphosource	https://doi.org/10.17602/M2/M5459 https://doi.org/10.17602/M2/M5470 https://doi.org/10.17602/M2/M48250 ark:/87602/m4/M103969 ark:/87602/m4/M31737 ark:/87602/m4/M81579 ark:/87602/m4/M83288 ark:/87602/m4/M103971 ark:/87602/m4/M104021 ark:/87602/m4/M81573
Hyracoid 3D models (m3)	Morphosource	ark:/87602/m4/M104159 ark:/87602/m4/M103971 ark:/87602/m4/M31737 ark:/87602/m4/M103969 ark:/87602/m4/M104021 ark:/87602/m4/M81573 ark:/87602/m4/M81579 ark:/87602/m4/M83288 https://doi.org/10.17602/M2/M5459 https://doi.org/10.17602/M2/M5470 https://doi.org/10.17602/M2/M48250
Supermatrix of 403 morphological characters	Cooper et al.^59^	
Comparative datasets used in our study	Mendeley data	https://doi.org/10.17632/dt8ws9s72j.1

**Software and algorithms**		

RevBayes v.1.1.0	https://revbayes.github.io/	
R v.4.3.1	https://cran.r-project.org/	

### Resource availability

#### Lead contact

Further information and requests for resources should be directed to and will be fulfilled by the lead contact, René Bobe (renebobe@gmail.com).

#### Materials availability

This study did not generate new unique reagents.

### Method details

#### Paleontological excavations

During the 2016-2019 field seasons, the Paleo-Primate Project Gorongosa discovered and documented seven paleontological localities with fossil vertebrates (GPL-1, GPL-2, GPL-6, GPL-7, GPL-8, GPL-11, and GPL-12), three additional localities with invertebrates only (GPL-3, GPL-9, and GPL-10), and two localities with *ex-situ* stone tools (GPL-4 and GPL-5). Menguere Hill, with abundant fossil wood, is the westernmost fossiliferous locality and it is not identified by a GPL number. These localities are listed in Table 1. Paleontological excavations with archaeological techniques were carried out at GPL-12 with the recovery of abundant *in situ* fossils. The team used a GPS unit Arrow Gold in conjunction with a total station to map the excavations and geology in the vicinity of GPL-12 (see Figure S1) and created a system of datums for future excavations. All excavated sediments were dry-sieved through a 3 mm mesh. Large, fragile fossils were plastered in blocks and carefully removed from the excavated area. Consecutive numbers called ‘lots’ captured changes in geology, stratigraphic breaks, and changes in a horizontal position within an excavation. The 3D coordinates (X, Y and Z) of complete fossils and identifiable fragments exposed through excavation were mapped with a Leica Builder-505 total station using EDM-Mobile software.^77^

#### Cosmogenic nuclides - atmospheric ^10^Be/^9^Be dating

For beryllium isotope analysis, ∼1 g of dry sediment was split from each sample. Be isotope analysis was performed at the CEREGE National Cosmogenic Nuclides Laboratory (LN2C) following the chemical updated separation procedure from Bourlès and colleagues.^31^^,^^78^^,^^79^^,^^80^ The natural authigenic ^9^Be concentrations were measured using the LN2C graphite-furnace Atomic Absorption Spectrophotometer (AAS) with a double beam correction (Thermo Scientific ICE 3400®). The authigenic ^10^Be concentrations were calculated using the spiked ^10^Be/^9^Be ratios normalized to the NIST 4325 Standard Reference Material [2.79 ± 0.03 x 10^11^],^81^ measured at the French AMS national facility ASTER, and decay-corrected using the ^10^Be half-life of 1.387 ± 0.012 Ma.^82^^,^^83^ The radioactive decay equation N_(t)_=N_0∗_ e^−λt^, where N_(t)_ is the authigenic ^10^Be/^9^Be ratio measured in the sample to date, N_0_ is the initial authigenic ^10^Be/^9^Be ratio, λ is the ^10^Be radioactive decay constant and t is the time elapsed since deposition was used to calculate the atmospheric ^10^Be ages.

#### Cosmogenic nuclides - ^26^Al/^10^Be dating

Based on the relative decay of ^26^Al and ^10^Be cosmogenic nuclides produced *in situ* in quartz (SiO_2_) minerals, the ^26^Al/^10^Be burial dating method^33^^,^^34^^,^^35^ can be applied to determine the burial duration of sedimentary deposits, provided that the strata are still buried a few meters below the modern erosion surface. Drawing on the results, burial durations can then be employed to deduce pre- and post-burial denudation rates in contexts for the time frame from 100 ka to ∼6 Ma.^36^ In the Gorongosa context, the method was used to constrain the burial duration for sections in the upper member of the Mazamba Formation beneath the modern erosion surface, and to explore the rates of pre- and post-burial denudation.

According to the Gorongosa Geological Map (Direcção Nacional de Geologia 2006, Folha 1834), sedimentary rocks assigned to the upper member of the Mazamba Formation crop out along the rift-shoulder cuesta in the southeastern portion of Gorongosa National Park as well as east and towards the northeast of the park (Figure 3). Rock samples, of which two were analyzed for their ^10^Be and ^26^Al isotope compositions (16-Gor-Muss-7 and 16-Gor-Muss-8), were collected from two detailed sedimentological sections measured from natural outcrops in the southeastern corner of the park at Mussapassua-Site-1 (680465.17°S, 7887565.19°E) and Mussapassua-Site-2 (681013.52°S, 7887909.36°E). For ^26^Al/^10^Be dating, one sample was selected from each section at respectively 15.0 and 10.5 m below the top, demarcated by the modern erosion surface. The sections are ∼650 m apart and in total between 14 to 17 m thick. They contain widely similar, well-correlated siliziclastic successions (chiefly consisting of coarse-grained quartz arenites overlain by interbedded sandstone and silt- to mudstone units towards the top of the section) that are preliminarily interpreted to record alluvial fan to fluvio-deltaic conditions. No fossils have been discovered in this region yet.

The physico-chemical preparations performed on the upper Mazamba Formation samples at CEREGE and the Accelerator Mass Spectrometry measurements of their ^10^Be and ^26^Al concentrations at ASTER (CEREGE, Aix-en-Provence) followed the method described in Lebatard et al. (2014).^33^ The obtained ^26^Al/^10^Be ratio of each sample allows for the determination of corresponding burial durations and the pre- and post-burial denudation rate experienced by the sediments using the methodology fully explained in ref. 35. The method relies on the parameters of Braucher and colleagues,^84^ and the respective half-life of ^26^Al (0.705 ± 0.024 Ma)^85^^,^^86^ and ^10^Be (1.387 ± 0.012 Ma).^82^^,^^83^ The computing process uses also the surface ^26^Al/^10^Be spallogenic production rate ratio of 6.61 ± 0.52 obtained from the normalization of the measured ^26^Al/^27^Al ratios to the in-house standard SM-Al-11, whose ^26^Al/^27^Al ratio of 7.401 ± 0.064 × 10^−12^ has been cross-calibrated^87^ against primary standards from a round-robin exercise.^88^ Using the CosmoCalc calculator (Version 1.8),^89^ the scaling factor was determined for the neutronic production rates^90^ and a sea level and high latitude (SLHL) production rate of 4.03 ± 0.18 at g^-1^ a^-1^.^91^^,^^92^ Minimum and maximum burial durations and before and after burial denudation rates are theoretically obtained by modeling of the ^10^Be and ^26^Al concentrations.^33^^,^^35^ In the model without post-burial production, no cosmogenic nuclides were accumulated in the samples while buried (infinite burial depth), which presumably results in a minimum burial duration. In the model with post-burial production, the samples are considered as remaining buried at their sampling depths and accumulated cosmogenic nuclides produced by muons, which presumably leads to maximum burial durations in a steady denudation over the burial period.^35^ Resulting from the propagation of uncertainties of the different parameters and measurements used during the computing, uncertainties associated with the ratios, the durations and the denudation rates are reported as 1σ.

Table S3 summarizes the results of all ^10^Be and ^26^Al measurements and derived ^26^Al/^10^Be ratios obtained from the two sediment samples from the upper Mazamba Formation. These data were used to compute the burial durations of the samples. A model of computation without post-burial production (Table S4) normally leads to a minimum burial duration. However, for sample 16-Gor-Muss-7, the model without post-burial production leads to a burial duration of 1.32 ± 0.54 Ma, while modeling with post-burial production, which usually results in maximum burial durations, yielded a burial duration of 971.99 ± 398.52 ka. For the second sample, 16-Gor-Muss-8, the computations using models without and with post-burial production led to similar results, revealing a minimum burial duration of 838.16 ± 220.96 ka and a maximum burial duration of 971.99 ± 256.24 ka, respectively. Thus, the two samples indicate a burial duration of ca. 1 Ma for both models. Thus, the upper member of the Mazamba Formation is of early Pleistocene age, at least for the studied part of the Mussapassua sections between 15 m and 10.5 m below the modern erosion surface.

For both models, high pre-burial denudation rates were obtained. Specifically for the model with post-burial production, a deduced pre-burial denudation rate of more than 1000 m.Ma^-1^ seems high, regarding that post-burial production represents more than 80% of the concentrations of the two cosmogenic nuclides. These high values of pre-burial denudation and the fact that there is still production even after burial below more than 10 m imply that there is probably no inheritance to consider. Considering post-burial denudation, a rate of 20.93 m.Ma^-1^ (Table S4) seems to fit the data best (i.e., it is coherent with the *in situ* observations) and is regarded as a reasonable value in the Urema Rift context.

#### Pedogenic carbonates

Stable carbon (δ^13^C) and oxygen (δ^18^O) isotope values of 17 pedogenic carbonates from GPL-1 were used to infer regional paleovegetation and climate patterns during the formation of the fossil bearing sediments. δ^13^C values serve as a robust and well-established tool to reconstruct past vegetation growing on the site following soil development.^93^ C_4_ photosynthesis is typically prevalent in warm and seasonally dry, open conditions with high light intensity, whereas the C_3_ pathway is advantageous under low water stress and at high-pCO_2_ conditions. Due to a difference in their discrimination against ^13^C during photosynthesis, δ^13^C values of most C_4_ plants range from -9 to -19 ‰, while those of C_3_ plants lie between -25 and -29 ‰, resulting in ^13^C/^12^C ratios of tropical grasses and sedges ca. 14 ‰ higher than most trees, shrubs, bushes, and herbaceous plants.^94^ The variability of δ^13^C in C_4_ plants can be attributed to three different C_4_ photosynthetic subpathways,^93^ while the variation in δ^13^C among C_3_ plants is affected by a variety of environmental factors including trophic effect, precipitation, temperature, drought, canopy density, salinity, light intensity, nutrient levels, and partial pressure of CO_2._^95^^,^^96^^,^^97^^,^^98^^,^^99^^,^^100^ Collectively, however, these effects on δ^13^C of C_3_ plants are still considerably small compared to the differences between C_3_ and C_4_ biomass. Pedogenic carbonate formed in equilibrium with soil-respired CO_2_ is typically enriched in ^13^C by 13.5 to 17.0 ‰ compared to the CO_2_ which respired from plants or was released during decomposition of soil organic carbon and related organic matter.^101^^,^^102^

Pedogenic carbonate forms in oxygen isotope equilibrium with soil water.^103^ The δ^18^O value of soil carbonate is a function of soil water composition and temperature. Soil water is derived from meteoric water, but can differ from this source water due to enrichment through evaporation from the soil surface, mixing with (evaporatively ^18^O-enriched) infiltrating water, and/or the addition of isotopically distinct water from overland and vadose zone flow.^104^ Nevertheless, δ^18^O values of modern pedogenic carbonate have a strong positive correlation with the composition of meteoric water, which in turn has a positive correlation with local air temperature.^105^ Collectively, this makes paleosol carbonate an important paleoclimate proxy. The composition of local meteoric water has a large influence on δ^18^O of soil water and hence pedogenic carbonate δ^18^O. Today, the climate of central Mozambique is a result of interactions between the African Monsoon, the Intertropical Convergence Zone, and the Zaire Air Boundary. These complex patterns complicate the comparison of absolute δ^18^O values of distant localities due to possibly different isotopic composition of local precipitation.

We sampled 17 pedogenic carbonate nodules for stable carbon and oxygen isotopic analysis (reported as δ^13^C and δ^18^O values) from section GPL-1NE. The nodules were cut in half and powder was extracted with a diamond tip drill from the center of the nodule. Stable isotope analysis was conducted at Goethe University and Senckenberg BiK-F Joint Stable Isotope Facility Frankfurt, Germany. We reacted 112 to 366 μg untreated powder with 99% H_3_PO_4_ for 90 min at 70°C in continuous flow mode using a Thermo MAT 253 mass spectrometer interfaced with a Thermo GasBench II. Analytical procedures follow.^106^ Carrara Marble with 2.01 ‰ VPDB (δ^13^C) and −1.74 ‰ VPDB (δ^18^O) was used as internal laboratory standard for calibration, as well as for determination of the carbonate content of each sample. Final isotopic ratios are reported against VPDB (δ^13^C) and VSMOW (δ^18^O); overall analytical uncertainties are better than 0.03 ‰ and 0.04 ‰, respectively.

#### Vertebrate paleontology

All fossil specimens are listed in the Paleo-Gorongosa Database, where each entry provides specimen number, locality, GPS coordinates, stratigraphic position, taxonomic attribution, and skeletal elements represented. Each specimen has the prefix PPG followed by the year of discovery, as in PPG2017-P-121. Following the prefix and year of discovery, the letter P refers to Paleontological collection (rather than archeological or osteological collections). Specimens were numbered sequentially as they were retrieved in the field each year. For the 2016-2019 field seasons, there are 678 specimens from the Mazamba Formation in the database. Many specimens are very fragmentary, but some are more complete and well-preserved teeth and skeletal elements. At all localities we collected all fossil specimens during surveys and excavations, even if the specimens were very fragmentary. Isolated teeth and tooth fragments are common across localities, with 147 specimens listed in the database. There are 10 mandibles or mandible fragments, at least 2 maxillary fragments, and 4 other cranial fragments. Postcranial elements and their fragments are the most common type of vertebrate fossil, with 436 specimens in the database. Mammals are the most abundant vertebrates across all localities, followed by turtles, crocodiles, sharks and batoids.

We used photogrammetry to build 3D models of several diagnostic fossils. All specimens are housed at the Paleontology Laboratory in Chitengo, Gorongosa National Park. Measurements were taken either from the 3D models or directly with sliding calipers in the lab.

Paleontological localities range in elevation from about 100 m to 120 m above current sea level (Figure 3). Excavations with the use of a total station to record the position of each specimen were carried out at GPL-12 and GPL-1. At both localities there are multiple fossil horizons exposed in the available sections (Figure 4). At GPL-12 (Facies 2) there is a high density of fossils that may constitute a bone bed, but further excavation is needed to assess its extent.

#### Morphometric analysis of chondrichthyes

We semi-automated the collection of teeth outlines, each defined by 100 equidistant semi-landmarks, by using a custom-written script that relies on the ‘jpeg’ 0.1-8.1^107^ and ‘geomorph’ 3.3.1^108^ R packages. Additionally, we created a script to transform the sample of shark teeth outlines provided in Türtscher et al.^43^ into a semi-landmark dataset compatible with our protocol. After combining the samples, three different datasets were generated: A) all 600 specimens, from four different genera; B) a subset of 547 specimens, with only *Galeocerdo* sp. and *Physogaleus* sp.; C) a subset of 436 individuals only with species of *Galeocerdo*: *G. aduncus, G. capellini, G. clarkensis, G. cuvier, G. eaglesomei,* and *G. mayumbensis*. In order to remove all differences due to translation, rotation and scale, we superimposed all the coordinates using a Generalized Procrustes analysis (GPA) algorithm.^109^ Then, the harmonic coefficients were extracted from the aligned 2D outlines using an elliptical Fourier transform (EFT), retaining >99% of harmonic power.^110^ Then we performed a Principal Component Analysis (PCA) of the harmonic coefficients to summarize shape variation. Thus, this protocol for outline analysis consisted of three steps 1) GPA, 2) EFT and 3) PCA, which were performed using the ‘Momocs’ 1.3.2 R package.^111^ Subsequently, a multi-group linear discriminant analysis (LDA) was performed to test if it was possible to distinguish among the different shark taxonomic groups and to classify the Gorongosa specimens into these categories. The LDA maximizes the separation between *a priori* defined groups. Since our number of original variables (i.e., harmonic coefficients,) exceeded the number of analyzed specimens, we carried out this analysis using the principal components (PCs) that accounted for 90% of the sample variance to reduce the dimensionality of the dataset. The LDA was carried out using the lda() function of the ‘MASS’ 7.3-51.6 R package.^112^ Performance was calculated using the confusion matrix from which the overall classification accuracy was computed, as well as the Cohen’s Kappa statistic.^113^^,^^114^ The complete dataset was resampled using a “leave-group-out” (LGOCV) cross-validation,^115^ as a way to assess classification performance. This cross-validation strategy generates multiple splits of the data into modelling and prediction sets. This process was carried out 200 times and the data were split into a modelling sub-set comprising 80% of randomly assigned observations, whereas the testing sub-set considered the remaining 20%. The number of repeats was chosen to get a consistent classification performance and to minimize uncertainty. The obtained cross-validated models were then used to classify the Gorongosa specimens into the taxonomic categories available by calculating their posterior probabilities. This analysis was repeated three times considering three different datasets as explained above.

#### Morphometric analysis of hyracoidea

In the Principal Component Analysis of the hyracoid left mandible PPG2018-P-1 (Figure 12), a GPA was performed on the landmark data to remove differences due to scale, translation, and rotation in order to obtain shape variables.^116^ This procedure was done using the gpagen() function available as part of the ‘geomorph’ R package 3.3.1.^117^ The semi-landmarks were slid on the models’ surface by minimizing Procrustes distance.^118^ This is an iterative process that works by allowing the semi-landmarks to slide along the surface to remove the effects of arbitrary spacing by optimizing the location of the semi-landmarks with respect to the consensus shape configuration. These obtained shape variables were then used in a principal component analysis (PCA) to summarize shape variation. The PCA was carried out using the gm.prcomp() function of the ‘geomorph’ R package 3.3.1.^117^

#### Phylogenetic analysis of hyracoidea

A Bayesian phylogenetic analysis of hyracoid species, combining morphological and stratigraphic range data from the fossil record was performed to infer hyracoid phylogenetic relationships using RevBayes v.1.1.0.^58^ The stratigraphic ranges are the first and last occurrences observed for a single species in the fossil record and were obtained from the Paleobiology Database (PBDB) https://paleobiodb.org/#/. For *Procavia capensis* (i.e., the only extant species under analysis), the minimum occurrence date was set to 0.0 Ma. We used a “Fossilized Birth Death Range Process” (FBDRP)^119^ prior on the tree topology, which allows us to incorporate stratigraphic information as part of our tree inference. We used an exponential prior of 10 to model both speciation (λ), and extinction (μ) rates. An extant sampling proportion (ρ) of 0.2 was used as not all living hyrax species were sampled whilst an exponential prior (ψ) of 10 was used to account for fossil sampling rate, and a uniform distribution between 56 and 66 Ma was used as a prior on origin time (φ). The morphological data came from^59^ and comprised a supermatrix of 403 morphological characters from where we extracted all the hyracoid species present. We collected all mandibular characters available in the Gorongosa hyracoid mandibles and added this information to the hyracoid morphological matrix (Mendeley data repository: https://doi.org/10.17632/dt8ws9s72j.1). The Mkv+Γ model^120^ was used for the morphological data, which was partitioned into unordered and ordered characters, and then further partitioned based on the maximum number of character states of each division. Possible ascertainment bias in the morphological matrix was considered by using RevBayes’ dynamic likelihood approach.^121^ An uncorrelated log-normal relaxed clock model with exponentially distributed hyperpriors (μ=2.0, σ^2^=3.0)^122^ was used for modelling branch rate variation among lineages for the morphological datasets. We performed the phylogenetic inference analysis using 10,000,000 Markov chain Monte Carlo (MCMC) generations. We visually inspected that the run achieved convergence and good mixing using trace plots, and that all parameters had an effective sample size >200 using the effectiveSize() function from the R package ‘coda’ v.0.19-4 in R v.4.0.2.^123^ After discarding a 25% burn-in we obtained a posterior distribution of 75,000 phylogenetic trees from which we computed a maximum a credibility tree (MCC) tree as a way of summarising our posterior tree sample (Figure 14). This MCC tree corresponds to the tree with the maximum product of the posterior clade probabilities. From this tree we also obtained divergence time estimates which are summarised in Table S9.

#### Body mass estimates

We estimated the body mass of the fossil hyracoids from Gorongosa, Mozambique using tooth dimensions from two molars. Regression equations were calculated using the perissodactyl-hyracoid model,^124^ as the Gorongosa hyracoids have a ‘perissodactyl-type’ of molar shape.^71^ Only m2 lengths were considered.^125^ Equations were fit by an ordinary least-squares criterion and lengths were log_10_ transformed. We used quasimaximum-likelihood estimates to compensate for detransformation bias.^126^ However, this method may also be inherently biased, so both ‘detransformed’ and ‘corrected’ values are reported.

**Table:** PPG2018-P-1: 19.61 mm

	Body mass kg	lower 95% CI	upper 95% CI
Detransformed masses	124.8411	124.3665	125.3157
Bias corrected masses	128.5548	128.0661	129.0435

**Table:** PPG2018-P-2: 20.77 mm

	Body mass kg	lower 95% CI	upper 95% CI
Detransformed masses	148.4171	147.9425	148.8916
Bias corrected masses	152.8321	152.3435	153.3208

### Quantification and statistical analysis

All statistical analyses were carried out using R v.4.3.1 https://cran.r-project.org/ Geometric morphometric analyses were performed using geomorph’ 3.3.1 (Adams & Otárola-Castillo^108^) and Momocs’ 1.3.2 R package (Bonhomme et al.^111^). Please refer to https://github.com/geomorphR/geomorph and https://momx.github.io/Momocs/articles/Momocs_intro.html for further details. The Bayesian phylogenetic analysis was carried out using RevBayes v.1.1.0^58^ based on this tutorial https://revbayes.github.io/tutorials/fbd/.

## Data Availability

Data Comparative datasets used in some of the fossil analyses can be found as a Mendeley data repository: https://doi.org/10.17632/dt8ws9s72j.1. This paper analyzes existing, publicly available data. These accession numbers/references for the datasets are listed in the key resources table. Code This paper does not report original code. Any additional information required to reanalyze the data reported in this paper is available from the lead contact upon request.
