# Supplementary material for: The first Miocene fossils from coastal woodlands in the southern East African Rift
Source: iScience. 2023 Aug 15;26(9):107644. doi: 10.1016/j.isci.2023.107644 (PMC10494320; doi:10.1016/j.isci.2023.107644)
Supplement: Document S1. Figure S1 and Tables S1–S9 [file mmc1.pdf]

iScience, Volume *26*

Supplemental information

The first Miocene fossils from coastal woodlands

in the southern East African Rift

René Bobe, Vera Aldeias, Zeresenay Alemseged, Robert L. Anemone, Will Archer, Georges Aumaître, Marion K. Bamford, Dora Biro, Didier L. Bourlès, Melissa Doyle Boyd, David R. Braun, Cristian Capelli, João d'Oliveira Coelho, Jörg M. Habermann, Jason J. Head, Karim Keddadouche, Kornelius Kupczik, Anne-Elisabeth Lebatard, Tina Lüdecke, Amélia Macôa, Felipe I. Martínez, Jacinto Mathe, Clara Mendes, Luis Meira Paulo, Maria Pinto, Darya Presnyakova, Thomas A. Püschel, Frederico Tátá Regala, Mark Sier, Maria Joana Ferreira da Silva, Marc Stalmans, and Susana Carvalho

Supplemental Information

Contents of this file
Figure S1
Tables S1 to S9

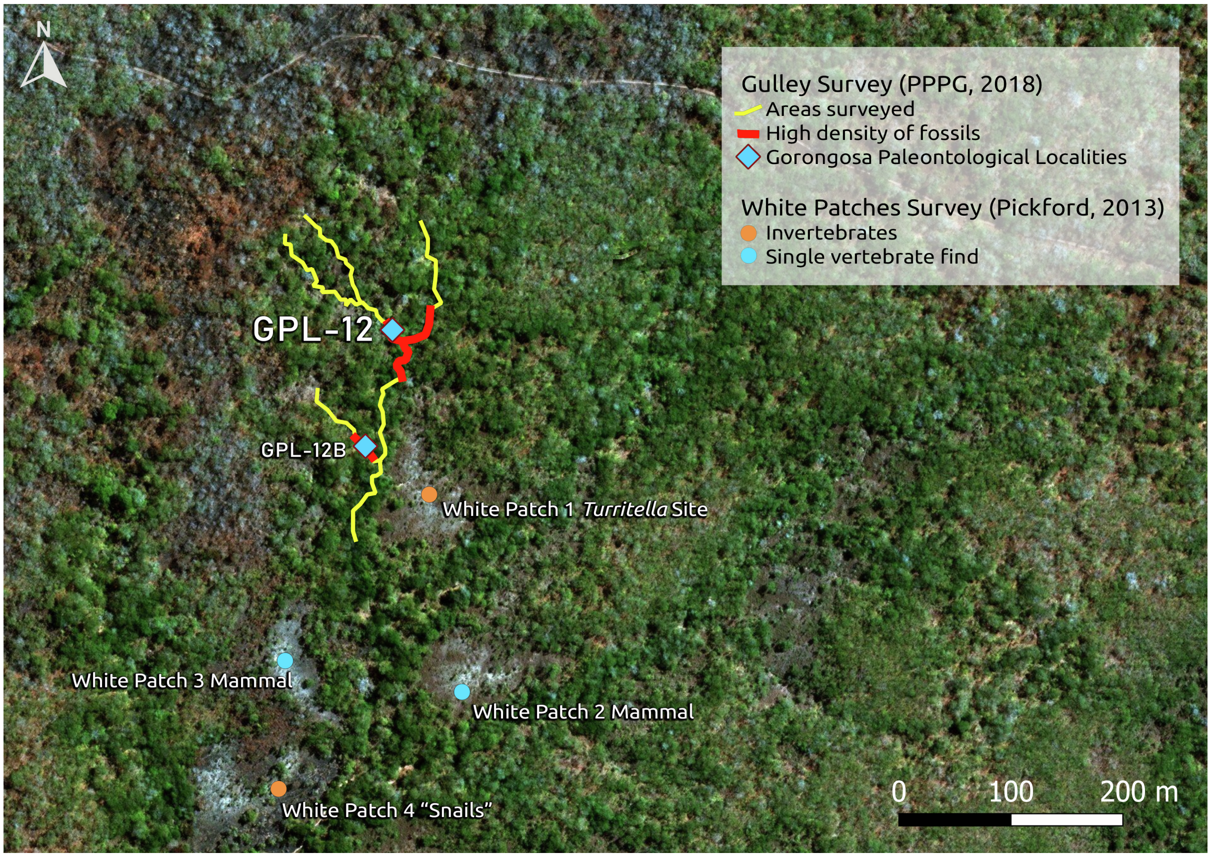

Figure S1. Map of the extent of GPL-12, and surrounding areas, related to Paleontological excavations (Methods Details).

Table S1. Sampling details for rock samples from the lower member of the Mazamba Formation (F=Fossil) and modern (M) sediment samples taken for atmospheric ^{10}Be dating.

Sample ID	Sample type	Locality/site	Decimal degree		Lithology	Sedimentary environment	Sampling depth below surface
			°S	°E			
Be18-Gor-GPL1NE-1	F	GPL1NE_section base	-18.9288	34.6476	coarse carbonate-cemented sandstone	fluvial, alluvial channel	excavated trench wall
Be18-Gor-GPL1NE-2	F	GPL1NE	-18.9288	34.6476	clay-bearing sandstone	fluvio-deltaic	excavated trench wall
Be18-Gor-GPL1NE-3	F	GPL1NE	-18.9288	34.6476	sandy claystone	estuarine, central basin/swamp	excavated trench wall
Be18-Gor-GPL1NE-4	F	GPL1NE	-18.9288	34.6476	clayey sandstone	fluvio-deltaic, distributary channel	excavated trench wall
Be18-Gor-GPL1NE-5	F	GPL1NE	-18.9288	34.6476	sandy claystone	estuarine, central basin/swamp	excavated trench wall
Be18-Gor-GPL1NE-6	F	GPL1NE_section top	-18.9288	34.6476	sandy clay- to marlstone	estuarine, central basin	excavated trench wall
Be18-Gor-GPL12-0.1	F	GPL12, STP101_section base	-18.9337	34.6456	coarse carbonate-cemented sandstone	fluvial, alluvial channel	excavated trench wall
Be18-Gor-GPL12-1.1	F	GPL12, STP101	-18.9337	34.6456	coarse carbonate-cemented reddish sandstone	fluvio-deltaic	excavated trench wall
Be18-Gor-GPL12-3.1	F	GPL12, STP101	-18.9337	34.6456	brown-beige sandy claystone	estuarine, central basin/swamp	excavated trench wall
Be18-Gor-GPL12-4.1	F	GPL12, STP101	-18.9337	34.6456	brown-beige clayey sandstone	fluvio-deltaic, distributary channel	excavated trench wall
Be18-Gor-GPL12-5.1	F	GPL12, STP101_section top	-18.9337	34.6456	olive-green claystone	estuarine, central basin	excavated trench wall
17-Gor-GPL6-8	F	GPL6 section base	-18.9271	34.6530	clayey sandstone	estuarine, central basin/swamp	excavated section wall
17-Gor-GPL6-3	F	GPL6 section center	-18.9271	34.6530	laminated claystone	estuarine, central basin/swamp	excavated section wall
17-Gor-GPL2-5	F	GPL2 section base	-18.9070	34.6757	clayey olive-brown sanstone	estuarine/marine, lagoonal shelf/barrier	excavated section wall
17-Gor-GPL2-10	F	GPL2 section top	-18.9070	34.6757	olive clayey sandstone	estuarine/marine, lagoonal shelf/barrier	excavated section wall
Be18-Gor-Pu-2	M	Pungwe River	-18.9944	34.3494	sandy clay	fluvial, alluvial floodplain c. 200 m north of river	60 cm, ca. 2-3 m above water level
Be18-Gor-Urem-1.1	M	Urema River	-18.9878	34.5693	dark-brown sandy	fluvial, riverbank	30 cm, at water level
Be18-Gor-Vun-1.1	M	Vunduzi River (?)	-18.4805	34.2103	muddy coarse sand	fluvial, riverbank	water-sediment interface at 1 cm water depth
Be18-Gor-VunS1-1.1	M	Stream S of Vunduzi River	-18.4899	34.2011	sandy mud	fluvial, riverbank	water-sediment interface at 1 cm water depth
Be18-Gor-Muc-1.1	M	Mucuro Mazi River	-18.5187	34.1915	muddy sand	fluvial, riverbank	water-sediment interface at 1 cm water depth
Be18-Gor-LUrem-1.1	M	Lake Urema	-18.9130	34.5178	muddy sand	fluvio-lacustrine,	30 cm, at water level
Be18-Bei-EstRi1-1	M	1st estuary NE Beira	-19.7873	34.9609	muddy sand	fluvio-deltaic, river-dominated estuary shore	20 cm, ca. 1 cm above water level
Be18-Bei-SavEst-1	M	Savane River estuary NE Beira	-19.6806	35.1396	organic-rich sandy mud/clay	lagoonal estuary shore on mangrove-dominated barrier peninsula	surface at water level
Be18-Bei-SavFor-1	M	Savane River estuary NE Beira	-19.6821	35.1399	organic-rich sandy mud/clay	mangrove forest/swamp on lagoonal barrier peninsula	20 cm, ca. 0.5 m above water level

Table S2. Concentrations of ^{10}Be and ^{9}Be and $^{10}Be/^{9}Be$ ratios for the Gorongosa samples, related to atmospheric ^{10}Be dating.

	Samples	Sample weight [g]	Measured (^{10}Be /^{9}Be) $*10^{-13}$	Authigenic ^{9}Be $* 10^{16}$ [at.g^{-1}]	Authigenic ^{10}Be $* 10^{7}$ [at.g^{-1}]	Authigenic ^{10}Be /^{9}Be $*10^{-8}$
Modern	Be18-Gor-Pu-2	0,9593	106,6344 ± 2,1964	5,1161 ± 0,1496	22,6219 ± 0,4653	4,4217 ± 0,3162
	Be18-Gor-Urem-1.1	0,9584	89,1654 ± 1,9579	7,9130 ± 0,2405	18,9476 ± 0,4153	2,3945 ± 0,1795
	Be18-Gor-Vun-1.1	0,9596	15,8407 ± 0,4884	1,6814 ± 0,0095	3,3464 ± 0,1021	1,9902 ± 0,1235
	Be18-Gor-VunS1-1.1	0,9574	86,4330 ± 1,8054	7,5941 ± 0,0724	18,3644 ± 0,3829	2,4182 ± 0,1109
	Be18-Gor-Muc-1.1	0,9585	84,7570 ± 2,1050	2,6621 ± 0,1283	17,9818 ± 0,4458	6,7548 ± 0,7323
	Be18-Gor-Lurem-1.1	0,9624	70,8797 ± 1,7002	3,3840 ± 0,1200	14,9327 ± 0,3574	4,4127 ± 0,3776
	Be18-Bei-EstRi1-1	0,9585	280,9860 ± 5,0466	4,2827 ± 0,0239	59,3875 ± 1,0660	13,8668 ± 0,5213
	Be18-Bei-SavEst-1	0,9583	77,6644 ± 1,8204	2,3125 ± 0,0795	16,4663 ± 0,3852	7,1206 ± 0,5920
	Be18-Bei-SavFor-1	0,9583	75,6599 ± 1,9046	2,6573 ± 0,0655	16,0133 ± 0,4023	6,0262 ± 0,4240
Lower Mazamba Formation	Be18-Gor-GPL1NE-1 (2)	0,9162	1,7757 ± 0,0005	0,0137 ± 0,0569	0,5941 ± 0,0186	0,1894 ± 0,0137
	Be18-Gor-GPL1NE-2	0,9174	0,1918 ± 0,0008	0,0011 ± 0,1369	0,0610 ± 0,0032	0,0096 ± 0,0011
	Be18-Gor-GPL1NE-3	0,9137	1,2693 ± 0,0005	0,0054 ± 0,1006	0,4189 ± 0,0137	0,0728 ± 0,0054
	Be18-Gor-GPL1NE-4 (2)	0,9158	0,0383 ± 0,0018	0,0011 ± 0,0457	0,0125 ± 0,0015	0,0045 ± 0,0011
	Be18-Gor-GPL1NE-5	0,9172	0,0082 ± 0,0024	0,0003 ± 0,0490	0,0027 ± 0,0005	0,0009 ± 0,0003
	Be18-Gor-GPL1NE-6 (2)	0,9151	2,6210 ± 0,0004	0,0196 ± 0,1535	0,8776 ± 0,0272	0,2065 ± 0,0196
	17-Gor-GPL2-5	0,9167	1,5327 ± 0,0005	0,0048 ± 0,0700	0,5178 ± 0,0162	0,0734 ± 0,0048
	17-Gor-GPL2-10 (2)	0,9174	1,9923 ± 0,0006	5,0197 ± 0,1192	0,6586 ± 0,0203	0,1312 ± 0,0102
	17-Gor-GPL6-3	0,9173	0,4146 ± 0,0006	0,0023 ± 0,0873	0,1396 ± 0,0056	0,0269 ± 0,0023
	17-Gor-GPL6-8	0,9192	0,1950 ± 0,0009	0,0040 ± 0,0568	0,0653 ± 0,0041	0,0296 ± 0,0040
	Be18-Gor-GPL12-0.1 (2)	0,9174	0,0464 ± 0,0015	0,0006 ± 0,1541	0,0156 ± 0,0016	0,0027 ± 0,0006
	Be18-Gor-GPL12-1.1	0,9174	0,0103 ± 0,0030	0,0003 ± 0,0754	0,0035 ± 0,0007	0,0008 ± 0,0003
	Be18-Gor-GPL12-3.1 (2)	0,9175	0,4907 ± 0,0006	0,0025 ± 0,0883	0,1649 ± 0,0071	0,0277 ± 0,0025
	Be18-Gor-GPL12-4.1	0,9191	0,0423 ± 0,0019	0,0008 ± 0,1172	0,0141 ± 0,0019	0,0030 ± 0,0008
	Be18-Gor-GPL12-5.1 (2)	0,9168	0,1879 ± 0,0009	0,0011 ± 0,0901	0,0630 ± 0,0037	0,0089 ± 0,0011

Table S3. Results of $^{26}Al/^{10}Be$ analyses, related to cosmogenic nuclide dating.

Sample	Depth (cm)	Depth ($g.cm^{-2}$)	Dissolved quartz (g)	^{9}Be carrier (10^{19} at.)	^{10}Be (10^{5} $at.g^{-1}$)			^{26}Al (10^{5} $at.g^{-1}$)			$^{26}Al/^{10}Be$		
16-Gor-Muss-7	1500	3750	20,1345	3,0244	9909,71	±	1328,88	37881,44	±	14645,17	3,8227	±	1,5642
16-Gor-Muss-8	1050	2625	20,1432	3,0511	11973,11	±	1589,53	57683,13	±	13054,15	4,8177	±	1,2640

Table S4. Model outputs of burial durations and denudation rates, related to cosmogenic nuclide dating.

Sample	Model Without Post-B production		Model With Post-B. production				
	Denud. before burial (m.Ma^{-1})	Min Burial duration (ka)	Denud. before B. (m.Ma^{-1})	Max Burial duration (ka)	Denud. after B. (m.Ma^{-1})	% [^{10}Be] Post-B.	% [^{26}Al] Post-B.
16-Gor-Muss-7	140,04	1 316,25 ± 539,66	1 054,85	971,99 ± 398,52	20,93	84	81
16-Gor-Muss-8	147,30	838,16 ± 220,96	1 746,90	971,99 ± 256,24	20,93	92	92

Table S5. List of fossil shark specimens used in the analysis of tooth outlines (n = 598), related to Figure 9. Cappetta 1970 refers to [1].

Specimen Number	n	Source/Museum	Locality	Epoch	Species	Reference
MPEG-1131-V	1	Aguilera et al. 2017	Brazil	Early Miocene	†*Carcharhinus ackermannii*	doi:10.1371/journal.pone.0182740
AMU-CURS-990	1	Carrillo-Briceño et al. 2016	Venezuela	Early Miocene	†*Carcharhinus brachyurus*	doi:10.5167/uzh-125933
MUN-STRI-43808	1	Carrillo-Briceño et al. 2019	Colombia	Middle Miocene	†*Carcharhinus gibbesii*	doi:10.5194/bg-16-33-2019
MPEG-1836-V	1	Aguilera et al. 2017	Brazil	Early Miocene	†*Carcharhinus perezi*	doi:10.1371/journal.pone.0182740
UAP-14.181-14	1	Andrianavalona et al. 2015	NW Madagascar	Miocene	†*Carcharhinus priscus*	doi:10.1371/journal.pone.0129444
UAP-13.159	1	Andrianavalona et al. 2015	NW Madagascar	Miocene	†*Carcharhinus* sp.	doi:10.1371/journal.pone.0129444
AMU-CURS-647	1	Carrillo-Briceño et al. 2016	Venezuela	Early Miocene	†*Galeocerdo aduncus*	doi:10.5167/uzh-125933
AMU-CURS-730	1	Carrillo-Briceño et al. 2016	Venezuela	Early Miocene	†*Galeocerdo aduncus*	doi:10.5167/uzh-125933
LPN 162	1	Capetta, 1970	France (Montpellier)	Miocene	†*Galeocerdo aduncus*	Cappetta 1970
LPN 163	1	Capetta, 1970	France (Montpellier)	Miocene	†*Galeocerdo aduncus*	Cappetta 1970
MUSM 3262	2	Landini et al. 2017	Peru	Late Miocene	†*Galeocerdo aduncus*	doi:10.1016/j.jsames.2016.12.010
NA	1	Pawellek et al. 2012	Libya	Early Pliocene	*Galeocerdo cuvier*	doi:10.1127/0077-7749/2012/0272
105Z100	1	Argyriou et al. 2015	Libya	Early Miocene	†*Galeocerdo mayumbensis*	doi:10.1016/j.jafrearsci.2014.11.008
112Z100	1	Argyriou et al. 2015	Libya	Early Miocene	†*Galeocerdo mayumbensis*	doi:10.1016/j.jafrearsci.2014.11.008
AMU-CURS-646	1	Carrillo-Briceño et al. 2016	Venezuela	Early Miocene	†*Galeocerdo mayumbensis*	doi:10.5167/uzh-125933
AMU-CURS-995	1	Carrillo-Briceño et al. 2016	Venezuela	Early Miocene	†*Galeocerdo mayumbensis*	doi:10.5167/uzh-125933
MPEG-1710-V	1	Aguilera et al. 2017	Brazil	Early Miocene	†*Galeocerdo mayumbensis*	doi:10.1371/journal.pone.0182740
MPEG-177-V	1	Aguilera et al. 2017	Brazil	Early Miocene	†*Galeocerdo mayumbensis*	doi:10.1371/journal.pone.0182740
MPEG-1854-V	1	Aguilera et al. 2017	Brazil	Early Miocene	†*Galeocerdo mayumbensis*	doi:10.1371/journal.pone.0182740
UAP-13.167	1	Andrianavalona et al. 2015	NW Madagascar	Miocene	†*Galeocerdo mayumbensis*	doi:10.1371/journal.pone.0129444
UAP-13.172	1	Andrianavalona et al. 2015	NW Madagascar	Miocene	†*Galeocerdo mayumbensis*	doi:10.1371/journal.pone.0129444
MPEG-781-V	1	Aguilera et al. 2017	Brazil	Early Miocene	†*Hemipristis serra*	doi:10.1371/journal.pone.0182740
MUN-STRI-34790	2	Carrillo-Briceño et al. 2019	Colombia	Middle Miocene	†*Hemipristis serra*	doi:10.5194/bg-16-33-2019
MUN-STRI-41132	1	Carrillo-Briceño et al. 2019	Colombia	Middle Miocene	†*Physogaleus contortus*	doi:10.5194/bg-16-33-2019
AMU-CURS-648	1	Carrillo-Briceño et al. 2016	Venezuela	Early Miocene	†*Physogaleus contortus*	doi:10.5167/uzh-125933
AMU-CURS-719	1	Carrillo-Briceño et al. 2016	Venezuela	Early Miocene	†*Physogaleus contortus*	doi:10.5167/uzh-125933
MUSM 3261	1	Landini et al. 2017	Peru	Late Miocene	†*Physogaleus contortus*	doi:10.1016/j.jsames.2016.12.010
EMRG-Chond-T-76	59	Vertebrate Collection, University of Vienna	USA (North Carolina)	Early Miocene	†*Galeocerdo aduncus*	doi:10.1017/pab.2021.6
NA	6	Haimuseum Aathal Switzerland	Germany	Miocene	†*Galeocerdo aduncus*	doi:10.1017/pab.2021.6
KM_DJ.033	3	Haimuseum Aathal Switzerland	Germany	Oligocene	†*Galeocerdo aduncus*	doi:10.1017/pab.2021.6
2012/0017/0240	14	Natural History Museum Vienna	USA (Florida)	Miocene	†*Galeocerdo aduncus*	doi:10.1017/pab.2021.6
UF 116830	2	Florida Museum of Natural History	USA (Florida)	Middle Miocene	†*Galeocerdo aduncus*	doi:10.1017/pab.2021.6
UF 217131	5	Florida Museum of Natural History	USA (Florida)	Middle Miocene	†*Galeocerdo aduncus*	doi:10.1017/pab.2021.6
UF 231021	1	Florida Museum of Natural History	USA (Florida)	Middle Miocene	†*Galeocerdo aduncus*	doi:10.1017/pab.2021.6
UF 232403	16	Florida Museum of Natural History	USA (Florida)	Middle Miocene	†*Galeocerdo aduncus*	doi:10.1017/pab.2021.6
UF 232425	1	Florida Museum of Natural History	USA (Florida)	Middle Miocene	†*Galeocerdo aduncus*	doi:10.1017/pab.2021.6
UF 234253	1	Florida Museum of Natural History	USA (Florida)	Middle Miocene	†*Galeocerdo aduncus*	doi:10.1017/pab.2021.6
UF 240484	7	Florida Museum of Natural History	USA (Florida)	Late Miocene	†*Galeocerdo aduncus*	doi:10.1017/pab.2021.6
UF 278633	1	Florida Museum of Natural History	USA (Florida)	Middle Miocene	†*Galeocerdo aduncus*	doi:10.1017/pab.2021.6
UF 28780	4	Florida Museum of Natural History	USA (Florida)	Middle Miocene	†*Galeocerdo aduncus*	doi:10.1017/pab.2021.6
UF/TRO 15237	1	Florida Museum of Natural History	USA (Florida)	Late Miocene	†*Galeocerdo aduncus*	doi:10.1017/pab.2021.6
24756	3	NMNH, Washington, D.C.	USA (North Carolina)	Miocene	†*Galeocerdo aduncus*	doi:10.1017/pab.2021.6
287850	27	NMNH, Washington, D.C.	USA (North Carolina)	Miocene	†*Galeocerdo aduncus*	doi:10.1017/pab.2021.6
KF-17E	1	Patnaik et al. 2014	India (Gujarat)	Early Miocene	†*Galeocerdo bigelowi*	doi:10.1017/pab.2021.6
NA	1	Lawley 1881	Italy (Tuscany)	Pliocene	†*Galeocerdo capellini*	doi:10.1017/pab.2021.6
IGM 5854	1	Universidad Nacional Autónoma de México	Mexico	Pliocene	†*Galeocerdo capellini*	doi:10.1017/pab.2021.6
1281	25	Universidad Nacional Autónoma de México	Mexico	Pliocene	†*Galeocerdo capellini*	doi:10.1017/pab.2021.6
S216(P49)	1	Müller 1999	USA (North Carolina)	Early Miocene	†*Galeocerdo casei*	doi:10.1017/pab.2021.6

S217(P49)	1	Müller 1999	USA (North Carolina)	Early Miocene	†*Galeocerdo casei*	doi:10.1017/pab.2021.6
P.30501	1	Natural History Museum London	USA (Alabama)	Early Eocene	†*Galeocerdo clarkensis*	doi:10.1017/pab.2021.6
P.30465-7	1	Natural History Museum London	USA (Alabama)	Early Eocene	†*Galeocerdo clarkensis*	doi:10.1017/pab.2021.6
NA	25	Haimuseum Aathal Switzerland	USA (South Carolina)	Late Eocene	†*Galeocerdo clarkensis*	doi:10.1017/pab.2021.6
UF 65552	1	Florida Museum of Natural History	USA (Florida)	Middle Miocene	*Galeocerdo cuvier*	doi:10.1017/pab.2021.6
NA	3	Haimuseum Aathal Switzerland	USA (Florida)	Miocene	*Galeocerdo cuvier*	doi:10.1017/pab.2021.6
NA	5	Haimuseum Aathal Switzerland	USA (North Carolina)	Late Miocene	*Galeocerdo cuvier*	doi:10.1017/pab.2021.6
NA	2	Florida Museum of Natural History	USA (North Carolina)	Pliocene	*Galeocerdo cuvier*	doi:10.1017/pab.2021.6
NA	1	Florida Museum of Natural History	USA (South Carolina)	Early Pliocene	*Galeocerdo cuvier*	doi:10.1017/pab.2021.6
NA	1	Florida Museum of Natural History	USA (North Carolina)	Miocene	*Galeocerdo cuvier*	doi:10.1017/pab.2021.6
EJ-XX-427	1	Florida Museum of Natural History	USA (Florida)	Miocene	*Galeocerdo cuvier*	doi:10.1017/pab.2021.6
UF/TRO 11181	1	Florida Museum of Natural History	USA (Florida)	Early Pliocene	*Galeocerdo cuvier*	doi:10.1017/pab.2021.6
UF/TRO 15482	3	Florida Museum of Natural History	USA (Florida)	Early Pliocene	*Galeocerdo cuvier*	doi:10.1017/pab.2021.6
UF/TRO 14220	3	Florida Museum of Natural History	USA (Florida)	Early Pliocene	*Galeocerdo cuvier*	doi:10.1017/pab.2021.6
UF 17860	2	Florida Museum of Natural History	USA (Florida)	Early Pliocene	*Galeocerdo cuvier*	doi:10.1017/pab.2021.6
UF 227304	1	Florida Museum of Natural History	USA (Florida)	Early Pliocene	*Galeocerdo cuvier*	doi:10.1017/pab.2021.6
UF 227889	1	Florida Museum of Natural History	USA (Florida)	Early Pliocene	*Galeocerdo cuvier*	doi:10.1017/pab.2021.6
UF 227871	2	Florida Museum of Natural History	USA (Florida)	Early Pliocene	*Galeocerdo cuvier*	doi:10.1017/pab.2021.6
UF 228801	1	Florida Museum of Natural History	USA (Florida)	Early Pliocene	*Galeocerdo cuvier*	doi:10.1017/pab.2021.6
UF/TRO 3801	3	Florida Museum of Natural History	USA (Florida)	Early Pliocene	*Galeocerdo cuvier*	doi:10.1017/pab.2021.6
UF/TRO 3876	3	Florida Museum of Natural History	USA (Florida)	Early Pliocene	*Galeocerdo cuvier*	doi:10.1017/pab.2021.6
UF/TRO 5438	4	Florida Museum of Natural History	USA (Florida)	Early Pliocene	*Galeocerdo cuvier*	doi:10.1017/pab.2021.6
UF/TRO 5603	1	Florida Museum of Natural History	USA (Florida)	Early Pliocene	*Galeocerdo cuvier*	doi:10.1017/pab.2021.6
UF/TRO 5679	1	Florida Museum of Natural History	USA (Florida)	Early Pliocene	*Galeocerdo cuvier*	doi:10.1017/pab.2021.6
UF/TRO 5700	2	Florida Museum of Natural History	USA (Florida)	Early Pliocene	*Galeocerdo cuvier*	doi:10.1017/pab.2021.6
UF/TRO 5745	2	Florida Museum of Natural History	USA (Florida)	Early Pliocene	*Galeocerdo cuvier*	doi:10.1017/pab.2021.6
UF/TRO 8604	1	Florida Museum of Natural History	USA (Florida)	Early Pliocene	*Galeocerdo cuvier*	doi:10.1017/pab.2021.6
UF/TRO 8935	1	Florida Museum of Natural History	USA (Florida)	Early Pliocene	*Galeocerdo cuvier*	doi:10.1017/pab.2021.6
UF/TRO 9227	2	Florida Museum of Natural History	USA (Florida)	Early Pliocene	*Galeocerdo cuvier*	doi:10.1017/pab.2021.6
EMRG-Chond-J-9	38	Vertebrate Collection, University of Vienna	Southeast Asia	Extant	*Galeocerdo cuvier*	doi:10.1017/pab.2021.6
EMRG-Chond-J-10	41	Vertebrate Collection, University of Vienna	Southeast Asia	Extant	*Galeocerdo cuvier*	doi:10.1017/pab.2021.6
EMRG-Chond-J-16	45	Vertebrate Collection, University of Vienna	nd	Extant	*Galeocerdo cuvier*	doi:10.1017/pab.2021.6
DGM 653-P	1	dos Reis 2005	Brasil (Pará)	Miocene	*Galeocerdo cuvier*	doi:10.1017/pab.2021.6
NA	1	Davis 1888	New Zealand	Middle Miocene	†*Galeocerdo davisi*	doi:10.1017/pab.2021.6
NA	14	Haimuseum Aathal Switzerland	Morocco (Ad Dakhla)	Late Eocene	†*Galeocerdo eaglesomei*	doi:10.1017/pab.2021.6
CGM 60025	1	Underwood et al. 2011	Egypt	Middle Eocene	†*Galeocerdo eaglesomei*	doi:10.1017/pab.2021.6
PAL 13577	1	Natural History Museum London	Nigeria	Middle Eocene	†*Galeocerdo eaglesomei*	doi:10.1017/pab.2021.6
P.13578	1	Natural History Museum London	Nigeria	Middle Eocene	†*Galeocerdo eaglesomei*	doi:10.1017/pab.2021.6
P.13579	1	Natural History Museum London	Nigeria	Middle Eocene	†*Galeocerdo eaglesomei*	doi:10.1017/pab.2021.6
P.13580	1	Natural History Museum London	Nigeria	Middle Eocene	†*Galeocerdo eaglesomei*	doi:10.1017/pab.2021.6
P.13581	1	Natural History Museum London	Nigeria	Middle Eocene	†*Galeocerdo eaglesomei*	doi:10.1017/pab.2021.6
P.13582	1	Natural History Museum London	Nigeria	Middle Eocene	†*Galeocerdo eaglesomei*	doi:10.1017/pab.2021.6
P.13583	1	Natural History Museum London	Nigeria	Middle Eocene	†*Galeocerdo eaglesomei*	doi:10.1017/pab.2021.6
P.13586	1	Natural History Museum London	Nigeria	Middle Eocene	†*Galeocerdo eaglesomei*	doi:10.1017/pab.2021.6
P.13588	1	Natural History Museum London	Nigeria	Middle Eocene	†*Galeocerdo eaglesomei*	doi:10.1017/pab.2021.6
P.13589	1	Natural History Museum London	Nigeria	Middle Eocene	†*Galeocerdo eaglesomei*	doi:10.1017/pab.2021.6
P.13591	1	Natural History Museum London	Nigeria	Middle Eocene	†*Galeocerdo eaglesomei*	doi:10.1017/pab.2021.6
P.13592	1	Natural History Museum London	Nigeria	Middle Eocene	†*Galeocerdo eaglesomei*	doi:10.1017/pab.2021.6
P.13593	1	Natural History Museum London	Nigeria	Middle Eocene	†*Galeocerdo eaglesomei*	doi:10.1017/pab.2021.6
P.73677	1	Natural History Museum London	Morocco (Ad Dakhla)	Late Eocene	†*Galeocerdo eaglesomei*	doi:10.1017/pab.2021.6
L.U. 211	1	Tewari et al. 1960	India (Gujarat)	Early Miocene	†*Galeocerdo gajensis*	doi:10.1017/pab.2021.6

DJ.033	1	Haimuseum Aathal Switzerland	USA (Texas)	Eocene	†*Galeocerdo latidens*	doi:10.1017/pab.2021.6
DJ.033	3	Haimuseum Aathal Switzerland	Great Britain	Eocene	†*Galeocerdo latidens*	doi:10.1017/pab.2021.6
NA	6	Haimuseum Aathal Switzerland	Togo	Late Eocene	†*Galeocerdo latidens*	doi:10.1017/pab.2021.6
NA	1	Haimuseum Aathal Switzerland	Morocco (Ad Dakhla)	Late Eocene	†*Galeocerdo latidens*	doi:10.1017/pab.2021.6
NA	3	Haimuseum Aathal Switzerland	UK (Sussex)	Eocene	†*Galeocerdo latidens*	doi:10.1017/pab.2021.6
	1	D. J. Kemp	UK (Hampshire)	Eocene	†*Galeocerdo latidens*	doi:10.1017/pab.2021.6
NA	2	D. J. Kemp	UK (Hampshire)	Eocene	†*Galeocerdo latidens*	doi:10.1017/pab.2021.6
DTK 14-19/9/85	2	D. J. Kemp	UK (Hampshire)	Eocene	†*Galeocerdo latidens*	doi:10.1017/pab.2021.6
DTK:2000.95.12.1	3	D. J. Kemp	UK (Hampshire)	Eocene	†*Galeocerdo latidens*	doi:10.1017/pab.2021.6
CGM 60026	1	Underwood et al. 2011	Egypt	Eocene	†*Galeocerdo latidens*	doi:10.1017/pab.2021.6
NA	10	Haimuseum Aathal Switzerland	USA (Florida)	Miocene	†*Galeocerdo mayumbensis*	doi:10.1017/pab.2021.6
UF 232399	4	Florida Museum of Natural History	USA (Florida)	Middle Miocene	†*Galeocerdo mayumbensis*	doi:10.1017/pab.2021.6
UF/TRO 6017	6	Florida Museum of Natural History	USA (Florida)	Middle Miocene	†*Galeocerdo mayumbensis*	doi:10.1017/pab.2021.6
UF/V 4963	1	Florida Museum of Natural History	USA (Florida)	Early Miocene	†*Galeocerdo mayumbensis*	doi:10.1017/pab.2021.6
UF/V 4988	1	Florida Museum of Natural History	USA (Florida)	Early Miocene	†*Galeocerdo mayumbensis*	doi:10.1017/pab.2021.6
UF/V 5118	1	Florida Museum of Natural History	USA (Florida)	Early Miocene	†*Galeocerdo mayumbensis*	doi:10.1017/pab.2021.6
UF/V 2676	1	Florida Museum of Natural History	USA (Florida)	Early Miocene	†*Galeocerdo mayumbensis*	doi:10.1017/pab.2021.6
UF/V 4181	2	Florida Museum of Natural History	USA (Florida)	Early Miocene	†*Galeocerdo mayumbensis*	doi:10.1017/pab.2021.6
NRPS-P12007	5	Swedish Museum of Natural History	New Guinea	Miocene	†*Galeocerdo mayumbensis*	doi:10.1017/pab.2021.6
N653-PDGM-DNPM	1	da Silva Santos & Travassos 1960	Brasil (Pará)	Early Miocene	†*Galeocerdo paulinoi*	doi:10.1017/pab.2021.6
334960	3	NMNH, Washington, D.C.	USA (Maryland)	Middle Miocene	†*Galeocerdo triqueter*	doi:10.1017/pab.2021.6
DJ.034	2	Haimuseum Aathal Switzerland	USA (Georgia)	Eocene	†*Hemipristis curvatus*	doi:10.1017/pab.2021.6
NA	3	Haimuseum Aathal Switzerland	Morocco (Ad Dakhla)	Late Eocene	†*Hemipristis curvatus*	doi:10.1017/pab.2021.6
NA	6	Haimuseum Aathal Switzerland	USA (Arkansas)	Early Pliocene	†*Hemipristis serra*	doi:10.1017/pab.2021.6
7-298	1	Haimuseum Aathal Switzerland	USA (California)	Miocene	†*Hemipristis serra*	doi:10.1017/pab.2021.6
NA	1	Leriche 1910	France (Paris Basin)	Oligocene early	†*Physogaleus acutus*	doi:10.1017/pab.2021.6
PAL366457	1	NMNH, Washington, D.C.	USA (Alabama)	Early Eocene	†*Physogaleus alabamensis*	doi:10.1017/pab.2021.6
5361/15	1	Malyshkina et al. 2013	Ukraine (Crimea)	Middle Eocene	†*Physogaleus alabamensis*	doi:10.1017/pab.2021.6
KM EZ-AF GA/Ca7-194	1	Haimuseum Aathal Switzerland	nd	Eocene	†*Physogaleus alabamensis*	doi:10.1017/pab.2021.6
EMRG-Chond-T-74	21	Vertebrate Collection, University of Vienna	Morocco (Ad Dakhla)	Eocene	†*Physogaleus alabamensis*	doi:10.1017/pab.2021.6
NA	2	Haimuseum Aathal Switzerland	USA (New Jersey)	Early Miocene	†*Physogaleus contortus*	doi:10.1017/pab.2021.6
EMRG-Chond-T-75	26	Vertebrate Collection, University of Vienna	USA (North Carolina)	Early Miocene	†*Physogaleus contortus*	doi:10.1017/pab.2021.6
P.9083-4	2	Natural History Museum London	Argentina	Late Oligocene	†*Physogaleus contortus*	doi:10.1017/pab.2021.6
NA	1	Florida Museum of Natural History	USA (Florida)	Miocene	†*Physogaleus contortus*	doi:10.1017/pab.2021.6
UF 227864	1	Florida Museum of Natural History	USA (Florida)	Early Pliocene	†*Physogaleus contortus*	doi:10.1017/pab.2021.6
UF 228424	1	Florida Museum of Natural History	USA (Florida)	Middle Miocene	†*Physogaleus contortus*	doi:10.1017/pab.2021.6
UF 231020	1	Florida Museum of Natural History	USA (Florida)	Middle Miocene	†*Physogaleus contortus*	doi:10.1017/pab.2021.6
UF 231225	3	Florida Museum of Natural History	USA (Florida)	Middle Miocene	†*Physogaleus contortus*	doi:10.1017/pab.2021.6
UF 240509	1	Florida Museum of Natural History	USA (Florida)	Late Miocene	†*Physogaleus contortus*	doi:10.1017/pab.2021.6
UF 28779	7	Florida Museum of Natural History	USA (Florida)	Middle Miocene	†*Physogaleus contortus*	doi:10.1017/pab.2021.6
UF/V 9977	1	Florida Museum of Natural History	USA (Florida)	Early Miocene	†*Physogaleus contortus*	doi:10.1017/pab.2021.6
UF/TRO 11514	1	Florida Museum of Natural History	USA (Florida)	Middle Miocene	†*Physogaleus contortus*	doi:10.1017/pab.2021.6
UF/TRO 14490	1	Florida Museum of Natural History	USA (Florida)	Middle Miocene	†*Physogaleus contortus*	doi:10.1017/pab.2021.6
UF/TRO 14631	3	Florida Museum of Natural History	USA (Florida)	Middle Miocene	†*Physogaleus contortus*	doi:10.1017/pab.2021.6
UF/TRO 15103	2	Florida Museum of Natural History	USA (Florida)	Middle Miocene	†*Physogaleus contortus*	doi:10.1017/pab.2021.6
UF/TRO 15236	1	Florida Museum of Natural History	USA (Florida)	Late Miocene	†*Physogaleus contortus*	doi:10.1017/pab.2021.6
UF/TRO 15241	1	Florida Museum of Natural History	USA (Florida)	Middle Miocene	†*Physogaleus contortus*	doi:10.1017/pab.2021.6
UF/TRO 15442	5	Florida Museum of Natural History	USA (Florida)	Middle Miocene	†*Physogaleus contortus*	doi:10.1017/pab.2021.6
UF/TRO 9881	2	Florida Museum of Natural History	USA (Florida)	Middle Miocene	†*Physogaleus contortus*	doi:10.1017/pab.2021.6

Table S6. Linear Discriminant Analysis of shark teeth, related to Figure 9.

a) LDA Shark genera (6 PCs)
Genera Confusion Matrix

	Carcharhinus	*Galeocerdo*	*Hemipristis*	*Physogaleus*
Carcharhinus	0.571428571	0	0.224489796	0
Galeocerdo	0	76.58843537	0.044217687	6.741496599
Hemipristis	0.108843537	0.360544218	0.880952381	0.197278912
Physogaleus	0	1.962585034	0.891156463	11.42857143

Accuracy	0.89
Cohen's Kappa	0.6652854

Gorongosa fossil predictions (posterior probabilities)	*Carcharhinus*	*Galeocerdo*	*Hemipristis*	*Physogaleus*
Gorongosa_A_PPG2019-P-129	0.00%	97.53%	0.02%	2.45%
Gorongosa_B_PPG2019-P-127	0.00%	96.04%	1.81%	2.16%

b) LDA using *Galeocerdo* & *Physogaleus* genera (4 PCs)
Galeocerdo & *Physogaleus* Confusion Matrix

	Galeocerdo aduncus	*Galeocerdo capellini*	*Galeocerdo clarkensis*	*Galeocerdo cuvier*	*Galeocerdo eaglesomei*	*Galeocerdo mayumbensis*	*Physogaleus alabamensis*	*Physogaleus contortus*
Galeocerdo aduncus	11.03759398	0.285714286	1.255639098	3.597744361	0.045112782	0	1.436090226	0.996240602
Galeocerdo capellini	0	0.116541353	0	0.165413534	0.007518797	0	0.011278195	0
Galeocerdo clarkensis	0.206766917	0.007518797	0.078947368	0.063909774	0.105263158	0	0.15037594	0
Galeocerdo cuvier	6.469924812	3.763157895	0.578947368	34.27067669	0.545112782	0.80075188	0.639097744	0.296992481
Galeocerdo eaglesomei	0.308270677	0.109022556	0.218045113	0.815789474	4.07518797	0.387218045	0.684210526	0
Galeocerdo mayumbensis	0	0.229323308	0.304511278	0.289473684	0.184210526	4.827067669	0	0
Physogaleus alabamensis	1.015037594	0	1.72556391	0.646616541	0.30075188	0	3.721804511	0.236842105
Physogaleus contortus	0.511278195	0	0.34962406	0	0	0	0.12406015	12.0037594

Accuracy	0.7022556
Cohen's Kappa	0.5994338

Gorongosa fossil predictions (posterior probabilities)	*Galeocerdo aduncus*	*Galeocerdo capellini*	*Galeocerdo clarkensis*	*Galeocerdo cuvier*	*Galeocerdo eaglesomei*	*Galeocerdo mayumbensis*	*Physogaleus alabamensis*	*Physogaleus contortus*
Gorongosa_A_PPG2019-P-129	12.36%	16.61%	11.22%	27.80%	13.72%	12.54%	5.75%	0.00%
Gorongosa_B_PPG2019-P-127	35.43%	1.01%	28.05%	8.16%	6.62%	0.19%	20.42%	0.12%

c) LDA using *Galeocerdo* species (4 PCs)
Galeocerdo species confusion Matrix

	Galeocerdo aduncus	*Galeocerdo capellini*	*Galeocerdo clarkensis*	*Galeocerdo cuvier*	*Galeocerdo eaglesomei*	*Galeocerdo mayumbensis*
Galeocerdo aduncus	17.06132075	0.429245283	3.382075472	3.844339623	0.023584906	0
Galeocerdo capellini	0	0.367924528	0.051886792	0.731132075	0.04245283	0
Galeocerdo clarkensis	0.273584906	0.018867925	0.54245283	0.113207547	0.533018868	0
Galeocerdo cuvier	6.806603774	4.443396226	0.981132075	44.33962264	0.768867925	1
Galeocerdo eaglesomei	0.386792453	0.113207547	0.235849057	0.716981132	4.985849057	0.872641509
Galeocerdo mayumbensis	0	0.287735849	0.466981132	0.254716981	0.25	5.674528302

Accuracy	0.729717
Cohen's Kappa	0.5749247

Gorongosa fossil predictions (posterior probabilities)	*Galeocerdo aduncus*	*Galeocerdo capellini*	*Galeocerdo clarkensis*	*Galeocerdo cuvier*	*Galeocerdo eaglesomei*	*Galeocerdo mayumbensis*
Gorongosa_A_PPG2019-P-129	9.43%	12.75%	11.14%	31.04%	18.08%	17.54%
Gorongosa_B_PPG2019-P-127	47.18%	0.11%	37.04%	2.52%	13.09%	0.07%

Confusion matrix entries are average cell counts across the 200 resamples

Table S7. List of fossil hyracoid mandibular specimens used in comparative sample, related to Figure 12.

Specimen	Museum	Online repository	Genus	Species	doi/ark
KA1−1190	Ditsong National Museum of Natural History	Morphosource	*Procavia*	*Procavia transvaalensis*	doi:10.17602/M2/M5459
G7052	Ditsong National Museum of Natural History	Morphosource	*Procavia*	*Procavia* sp.	doi:10.17602/M2/M5470
H.5281.B	University Museum of Zoology, Cambridge	Morphosource	*Dendrohyrax*	*Dendrohyrax arboreus*	doi:10.17602/M2/M48250
RU18568	National Museums of Kenya		*Afrohyrax*	*Afrohyrax* sp.	
ZP349	National Museums of Kenya		*Afrohyrax*	*Afrohyrax championi*	
RU15198(A)	National Museums of Kenya		*Afrohyrax*	*Afrohyrax championi*	
DPC2150	Duke Lemur Center Division of Fossil Primates	Morphosource	*Saghatherium*	*Saghatherium humarum*	ark:/87602/m4/M103969
DPC18145	Duke Lemur Center Division of Fossil Primates	Morphosource	*Saghatherium*	*Saghatherium bowni*	ark:/87602/m4/M31737
DPC17675	Duke Lemur Center Division of Fossil Primates	Morphosource	*Thyrohyrax*	*Thyrohyrax meyeri*	ark:/87602/m4/M81579
DPC13282	Duke Lemur Center Division of Fossil Primates	Morphosource	*Saghatherium*	*Saghatherium bowni*	ark:/87602/m4/M83288
DPC2763	Duke Lemur Center Division of Fossil Primates	Morphosource	*Thyrohyrax*	*Thyrohyrax domorictus*	ark:/87602/m4/M103971
DPC15384	Duke Lemur Center Division of Fossil Primates	Morphosource	*Saghatherium*	*Saghatherium bowni*	
DPC5283	Duke Lemur Center Division of Fossil Primates	Morphosource	*Megalohyrax*	*Megalohyrax eocaenus*	ark:/87602/m4/M104021
DPC12048	Duke Lemur Center Division of Fossil Primates	Morphosource	*Saghatherium*	*Saghatherium bowni*	ark:/87602/m4/M81573

Table S8. List of fossil hyracoid lower third molars used in comparative sample, related to Figure 13.

Specimen	Museum	Online	Taxon	doi/ark notes
ZP1508	NMK		*Bunohyrax* aff. *fajumensis*	cast
RU15198(A)	NMK		*Afrohyrax championi*	
DPC7369	Duke Lemur Center	Morphosource	*Thyrohyrax domorictus*	ark:/87602/m4/M104159
RU18568	NMK		*Afrohyrax* sp.	
ZP349	NMK		*Afrohyrax championi*	cast
ZP347	NMK		*Afrohyrax championi*	cast
ZP1211	NMK		*Thyrohyrax domorictus*	cast
WK18206(A)	NMK		*Afrohyrax championi*	
DPC2763	Duke Lemur Center	Morphosource	*Thyrohyrax domorictus*	ark:/87602/m4/M103971
DPC18145	Duke Lemur Center	Morphosource	*Saghatherium bowni*	ark:/87602/m4/M31737
DPC2150	Duke Lemur Center	Morphosource	*Saghatherium humarum*	ark:/87602/m4/M103969
DPC5283	Duke Lemur Center	Morphosource	*Megalohyrax eocaenus*	ark:/87602/m4/M104021
DPC12048	Duke Lemur Center	Morphosource	*Saghatherium bowni*	ark:/87602/m4/M81573
NW22558 (C)	NMK		*Meroehyrax kyongoi*	
DPC17675	Duke Lemur Center	Morphosource	*Thyrohyrax meyeri*	ark:/87602/m4/M81579
DPC15384	Duke Lemur Center	Morphosource	*Saghatherium bowni*	
DPC13282	Duke Lemur Center	Morphosource	*Saghatherium bowni*	ark:/87602/m4/M83288
ZP1255	NMK		*Parapliohyrax mirabilis*	cast
BN802 (H)	NMK		*Parapliohyrax ngororaensis*	
LP22529	NMK		*Thyrohyrax microdon*	
KA1−1190	Ditsong NMNH	Morphosource	*Procavia transvaalensis*	doi:10.17602/M2/M5459
G7052	Ditsong NMNH	Morphosource	*Procavia* sp.	doi:10.17602/M2/M5470
NK41304	NMK		*Dendrohyrax* cf. *validus*	
NK36934	NMK		*Dendrohyrax* cf. *validus*	
H.5281.B	University Museum of Zoology, Cambridge	Morphosource	*Dendrohyrax arboreus*	doi:10.17602/M2/M48250

Table S9. Divergence time estimates and posterior support for the hyracoid phylogeny, related to Figure 14.

Node Number	Minimum bound for the Age 95% highest posterior density interval (HPD) [Ma]	Maximum bounds for the Age 95% highest posterior density interval (HPD) [Ma]	Mean divergence time [Ma]	Posterior support
1	12	23	17.5	0.4
2	16	26.6	21.3	0.72
3	28.1	32.1	30.1	0.74
4	33.9	36.1	35	0.68
5	34.1	38.4	36.3	0.74
6	35.1	41	38	0.29
7	33.9	35.8	34.9	0.9
8	34.1	37	35.5	0.65
9	33.9	35.7	34.8	0.98
10	34.7	38.7	36.7	0.98
11	33.9	37.4	35.7	0.92
12	35.6	40.9	38.3	0.82
13	28.2	34.7	31.4	0.4
14	33.9	36.7	35.3	0.42
15	33.9	35.5	34.7	0.99
16	34.4	38.7	36.5	0.81
17	36.3	42.2	39.3	0.25
18	37	43.6	40.3	0.21
19	33.9	36	35	0.68
20	34	38	36	1
21	37.7	45	41.4	1
22	39.4	47.6	43.5	0.49
23	41.8	49.9	45.8	1
24	47.8	54.8	51.3	0.92
25	56	59.6	57.8	1

Supplemental References

[1] Cappetta, H. (1970). Les Sélaciens du Miocène de la région de Montpellier. Palaeovertebrata *Mémoire extraordinaire*, 1-139.
